# Microglia through MFG-E8 signaling decrease the density of degenerating neurons and protect the brain from the development of cortical infarction after stroke

**DOI:** 10.1371/journal.pone.0308464

**Published:** 2024-08-07

**Authors:** Eric Yuhsiang Wang, Hank Szuhan Chen, Meng-Chih Wu, Ya Lan Yang, Hwai-Lee Wang, Che-Wei Liu, Ted Weita Lai

**Affiliations:** 1 Graduate Institute of Biomedical Sciences, China Medical University, Taichung, Taiwan; 2 School of Medicine, China Medical University, Taichung, Taiwan; 3 School of Chinese Medicine, China Medical University, Taichung, Taiwan; 4 Graduate Institute of Clinical Medical Science, China Medical University, Taichung, Taiwan; 5 Department of Plastic and Reconstructive Surgery, China Medical University Hospital, Taichung, Taiwan; 6 Neuroscience and Brain Disease Center, China Medical University, Taichung, Taiwan; 7 Drug Development Center, China Medical University, Taichung, Taiwan; 8 Translational Medicine Research Center, China Medical University Hospital, Taichung, Taiwan; Albany Medical College, UNITED STATES OF AMERICA

## Abstract

Neuronal loss is a hallmark of stroke and other neurodegenerative diseases, and as such, neuronal loss caused by microglia has been thought to be a contributing factor to disease progression. Here, we show that microglia indeed contribute significantly to neuronal loss in a mouse model of stroke, but this microglial-dependent process of neuronal clearance specifically targets stressed and degenerating neurons in the ischemic cortical region and not healthy non-ischemic neurons. Nonspecific stimulation of microglia decreased the density of neurons in the ischemic cortical region, whereas specific inhibition of MFG-E8 signaling, which is required for microglial phagocytosis of neurons, had the opposite effect. In both scenarios, the effects were microglia specific, as the same treatments had no effect in mice whose microglia were depleted prior to stroke. Finally, even though the inhibition of MFG-E8 signaling increased neuronal density in the ischemic brain region, it substantially exacerbated the development of cortical infarction. In conclusion, microglia through MFG-E8 signaling contribute to the loss of ischemic neurons and, in doing so, minimize the development of cortical infarction after stroke.

## Introduction

As one of the primary phagocytes in the brain, microglia play key roles in the clearance of neurons [1] and cell debris [2–4] and in the pruning of synapses throughout development and in neurological diseases [5–10]. Furthermore, several findings have supported the idea that microglial phagocytosis of neurons is a major contributing factor to neuronal loss and overall disease progression in neurodegenerative diseases. For example, inflammatory stimuli such as amyloid β or bacterial ligands, including lipoteichoic acid and lipopolysaccharide (LPS), triggered microglial phagocytosis of otherwise viable neurons in primary rat cultures of neurons and glia *in vitro*, and preventing microglial phagocytosis with blockers of phosphatidylserine, milk-fat globule epidermal growth factor-8 (MFG-E8), or MFG-E8 receptor or knockout of MFG-E8 in culture protected these neurons from phagocytic loss [4, 11–14]. In addition, disruption of phagocytosis in a non-cell-type-specific manner by injection of an MFG-E8 receptor inhibitor or a complement inhibitor or global genetic knockout of MFG-E8, Mer receptor tyrosine kinase, or P2Y_6_ receptor minimized neuronal loss and disease progression in rodent models of neurodegenerative diseases [13–16]. Nevertheless, contrary to the evidence suggesting a causative role of microglial phagocytosis in neurodegeneration, microglia have been reported to be neuroprotective against neurodegenerative diseases in other studies. For instance, intravascular injection of exogenous microglia minimized brain injury in gerbils subjected to global cerebral ischemia [17], whereas selective ablation of microglia by colony-stimulating factor 1 receptor (CSF1R) inhibition exacerbated brain infarction in mice subjected to focal ischemic stroke and repopulation of microglia reversed the effect of microglial ablation [18–20].

The reason for the apparent contradiction between the detrimental effect of microglial phagocytosis in neurodegeneration and the overall beneficial role of microglia in disease outcome remains elusive. One possible explanation is that, in addition to microglia, other cell types in the brain, including astrocytes, also exhibit phagocytic activities, and their phagocytic efficiency and priorities may be different than those of microglia [3, 21–23]. Therefore, while global inhibition of phagocytosis in a non-specific manner could offer therapeutic benefits in disease models [13–15], these effects could have resulted from inhibition of phagocytosis by non-microglial cell types. Another possible explanation is that microglia can exhibit non-phagocytic activities that are neuroprotective, so much so that they can overturn deleterious effects of phagocytosis. Indeed, microglia, in part through their release of soluble neurogenic factors, have been shown to be crucial in the promotion and guidance of neurogenesis [24–29], which in turn could have contributed to microglia-mediated neuroprotection in neurodegenerative disease models. Finally, one cannot rule out the possibility that the observation of neuronal loss in cell cultures and in animal models of neurodegenerative diseases could be protective rather than pathogenic to overall disease outcome.

Interestingly, it has historically been postulated that microglia-mediated removal of injured or stressed neurons could serve as a mean to prevent spillover of neurotoxic cell debris during neurodegeneration and therefore could be beneficial to brain recovery [2, 30]. Nevertheless, there has been little or no direct evidence to support this hypothesis. In the present study, we investigated whether microglia contribute to neuronal loss after stroke and how such neuronal loss affects the development of cortical infarction. Our data showed that microglia through MFG-E8 signaling, which is required for microglial phagocytosis of neurons, is indeed a significant contributor to neuronal loss in the ischemic cortical region, but this process has no effect on the density of healthy non-ischemic neurons. Importantly, this process, by which ischemic and degenerating neurons are selectively removed, protects the brain against the development of cortical infarction.

## Materials and methods

### Mice and diet

Male C57BL/6 mice (8–12 weeks old; 21–30 g) were used for the *in vivo* experiments, and neonatal mice (E14–E16) were used to prepare the primary cortical cultures. The mice were purchased from the National Laboratory Animal Center (Taipei, Taiwan) and were housed in ventilated cages with food and water *ad libitum*. To deplete microglia from the mouse brain, the mouse diet was replaced with a PLX3397-containing diet (1000 mg PLX3397 per kg of AIN-76A diet, prepared as described previously [31]) for 7 days (or 3 or 21 days, as indicated). For mice without depletion of microglia, the mouse diet was replaced with a PLX3397-lacking AIN-76A diet, which is referred to as the control diet in this manuscript. All experiments were conducted in accordance to the ARRIVE guidelines and the Institutional Guidelines of the China Medical University for the Care and Use of Experimental Animals (IGCMU-CUEA) and approved by the Institutional Animal Care and Use Committee (IACUC) of the China Medical University (Taichung, Taiwan) (Protocol No. CMUIACUC-2021-348).

### Mouse model of stroke and drug treatments

Transient ischemic stroke was induced by 2-h distal middle cerebral arterial occlusion (dMCAo) in mice as described previously [32]. Briefly, under isoflurane anesthesia, a burr hole was drilled on the right temporal bone covering the distal branch of the middle cerebral artery, which was then ligated by a 10–0 nylon suture. To facilitate the development of ischemia, the right common carotid artery was also exposed through a skin incision, isolated from the vagus nerve, and ligated with a cotton thread. During the 2-h occlusion period, the cranial window was closed, and body temperature was kept warm on a heating pad. Thereafter, all ligations were removed to allow for full reperfusion, and all surgical wounds were closed with 6–0 nylon sutures. For experiments in which microglial phagocytosis was stimulated, mice were injected with LPS (0.2 mg/kg, i.p.; from *E*. *coli* serotype O111:B4, Sigma Aldrich) or saline 48 h prior to dMCAo. Mice subjected to LPS treatment would initially show mild depressive-like behaviors, including body weight loss, anhedonia, and decreased locomotion, but the symptoms typically resolve by 48 h post-treatment. For experiments in which microglial phagocytosis was inhibited, mice were infused with cRGD (1 mM x 5 μl at 1 μl/min, i.c.v.; Bachem) or PBS into the contralateral (left, non-ischemic) ventricle (stereotaxic coordinates: AP: -0.2 mm, ML: 1.0 mm, DV: 2.25 mm) via a fine needle (Hamilton; Model 1701RN) immediately prior to dMCAo.

### Primary cortical cultures

Dissociated primary cultures were isolated and cultured as described previously [33, 34]. Briefly, under isoflurane anesthesia, a transverse incision was made on the lower abdominal region of the pregnant female mice to expose the uterus and embryos. Mouse embryos (E16-18) were then transferred to a petri dish filled with ice-cold PBS, and their scalp and dura were gently removed by fine forceps. The cortices were quickly isolated and immersed in a 1:1 Neurobasal:DMEM containing 2% B27, 0.5 mM L-glutamine, and 1% penicillin/streptomycin, and after gentle dissociation by means of pipetting, the dissociated cells were centrifuged (1200 rpm x 5 min) to have the supernatant replaced with fresh medium. The dissociated cells (3 x 10^5^ cells per well) were then seeded onto poly-D-lysine-coated glass in 24-well plates. Each well had 500 μl of medium, half of which was changed 3 days after seeding, and was treated with either PBS or LPS (0.1 μg/ml) with or without cRGD (50 μM) on the 7^th^ day for 24 h to induce or inhibit microglial phagocytosis. The medium was collected for ELISA (R&D Systems, cat. # DY406–05 and DY410–05), and the cell samples were fixed with 4% paraformaldehyde for immunofluorescence staining.

### Immunofluorescence and Fluoro-Jade B (FJ) staining

Immunofluorescence staining of 4% paraformaldehyde-fixed coronal brain slices (25 μm in thickness) and primary cortical cultures for microglia and neurons was performed with primary antibodies against Iba1 (1:500, 4°C overnight; Genetex, cat. # GTX100042) and NeuN (1:500, 4°C overnight; Abcam, cat. # MAB377), respectively, and with the corresponding secondary antibodies (1:800 anti-mouse 596, 1 h at room temperature; Abcam, cat. # ab150116; 1:800 anti-rabbit 488, 1 h at room temperature; Abcam, cat. # ab150077), as described previously [35]. In a control experiment, omitting the primary antibodies in our staining protocol showed a lack of false positive signal from staining with only the secondary antibodies (data not shown). To quantify the density of degenerating neurons, a subset of 4% paraformaldehyde-fixed coronal brain slices (25 μm in thickness) was mounted onto glass slides and stained with FJ (Merck, cat. # AG310-30MG) as described previously [36]. All images were captured using a fluorescence microscope (Revolve 4, Echo) and analyzed using the image analysis software ImageJ (NIH) by investigators blinded to the treatment groups.

### Determination of cortical infarction and blood-brain barrier (BBB) disruption

To quantify cortical infarction volume, mice were euthanized by an overdose of urethane (i.p.) 24 h after dMCAo. After the mice were perfused with normal saline, the mouse brains were collected, sectioned into 2-mm thick coronal brain sections, and stained with 2% triphenyltetrazolium chloride (TTC; Sigma Aldrich, cat. # T8877) at 37°C for 10 min. The areas of cortical infarction (in mm^2^) were measured using the image analysis software ImageJ (NIH, USA); thereafter, the volumes of cortical infarction (in mm^3^) were calculated by multiplying the areas of cortical infarction by the thickness of each coronal section (2 mm). To quantify the severity of BBB disruption, mice were subjected to unilateral craniectomy (diameter of 1 mm) above the right motor cortex or stroke induced by dMCAo, both of which were previously shown to cause unilateral BBB disruption [37], and 24 h thereafter, the mice were euthanized by an overdose of urethane (i.p.). The mouse brains were isolated and sectioned coronally (2 mm in thickness), and the right and left cortices were collected from the brain section that was located +2 to +4 mm anterior to lambda. BBB permeability, indicated by the amount of albumin that was extravasated into each cortex, was determined by western blotting as described previously. Primary antibodies against albumin (1:5000; Abcam, cat. # ab106582) and ɑ-tubulin (1:5000; Genetex, cat. # GTX628802) were used.

### Randomization and exclusion

Mice in different treatment groups were randomized with the RAND-BETWEEN function in Microsoft Excel and were blinded to investigators carrying out surgery, immunofluorescence and FJ staining, and cell counting. For mice subjected to stroke induced by dMCAo, the mortality rate was 9 out of 50 mice in the first 24 h and was equally distributed between the control and test treatments. Mice that died during this period had to be excluded due to tissue necrosis. In the neurogenesis experiment, one mouse died immediately after an intraperitoneal injection, likely due to accidental puncture of a large vessel due to technical incompetence. Moreover, two mouse brain samples from mice treated with PLX3397 and LPS were accidentally lost prior to immunofluorescence staining and analysis when our freezers were moved to a new laboratory location. To allow for sufficient power for statistical analysis, data that were considered outliers, specifically defined as those that were more than 2.4 standard deviations from the mean, were excluded from data presentation and analysis. Only one data point in this study fit our strict criteria for an outlier.

### Data presentation and statistical analysis

Except for the excluded data outlined above, all data are presented as individual data points and the mean ± sem. Comparisons between two groups were by unpaired t test, and comparisons among three groups were by 1-way ANOVA followed by Tukey’s multiple comparisons test. In addition, comparisons between the treatment groups for the neurogenesis experiments were by 2-way ANOVA, and the comparisons between different brain regions and treatment groups in the BBB experiments were by 2-way repeated-measures ANOVA, matching brain regions from the same mice, followed by Holm-Sidak’s multiple comparisons test.

## Results

### Depletion of microglia increased the density of neurons in the ischemic cortical region after stroke

**Fig 1:**
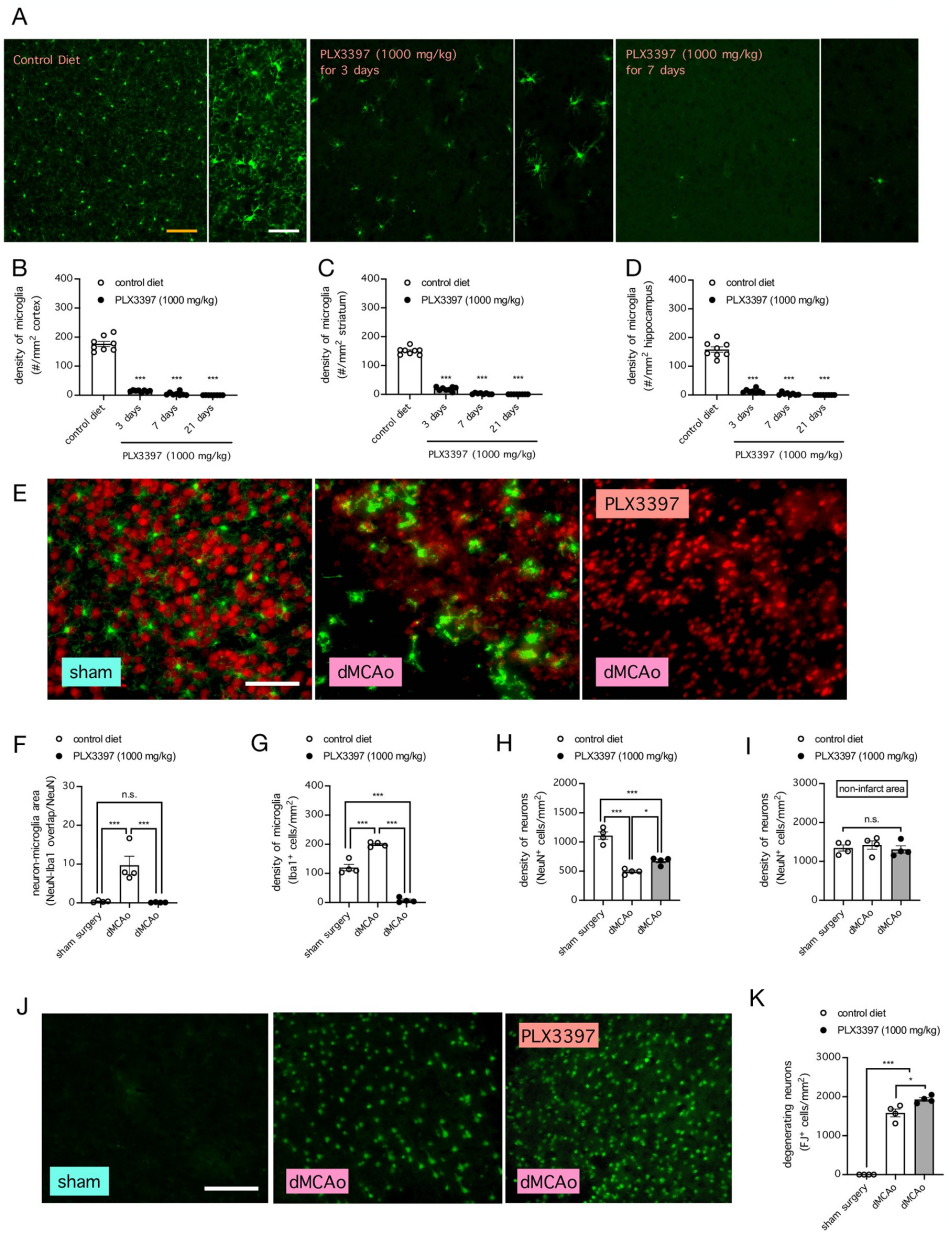
Colony-stimulating factor 1 receptor (CSF1R) inhibition depleted microglia and increased the density of neurons in the ischemic cortical region after stroke. ***A***, Representative images showing the density of microglia (Iba1^+^ cells) residing in the motor cortex of mice fed with control diet or a diet containing PLX3397 (1000 mg/kg of rodent chow) for 3 or 7 days. No microglia could be found in the motor cortex when the mice were fed with the PLX3397-containing diet for 21 days. Bars indicate 100 μm (*orange*) and 50 μm (*white*). ***B***, Summarized data for **A**. n = 8 mice per group; *** *P* < 0.001 when compared by 1-way ANOVA followed by Tukey’s multiple comparisons test. ***C-D***, Same as **B**, except for the striatum (**C**) and hippocampus (**D**). *** *P* < 0.001 when compared by 1-way ANOVA followed by Tukey’s multiple comparisons test. ***E***, Representative images showing neurons (NeuN^+^ cells; *red*) and microglia (Iba1^+^ cells; *green*) in the motor cortex of mice subjected to sham surgery or ischemic stroke induced by distal middle cerebral arterial occlusion (dMCAo) with or without microglial ablation by PLX3397 (1000 mg/kg of rodent chow for 7 days). Bar indicates 90 μm. ***F-H***, Summarized data for **E**, showing overlap between NeuN and Iba1 (**F**), the density of microglia (Iba1^+^ cells) (**G**), and the density of neurons (NeuN^+^ cells) (**H**) in the ischemic/sham area of the cortex. n = 4 mice per group; * *P* < 0.05 and *** *P* < 0.001 when compared by 1-way ANOVA followed by Tukey’s multiple comparisons test. n.s. indicates no significant difference. ***I***, Same as **H**, but in the contralateral area of the cortex. ***J***, Representative images showing degenerating neurons (Fluoro-Jade B^+^ (FJ^+^) cells) in the motor cortex of mice subjected to ischemic stroke induced by dMCAo with or without microglial ablation by PLX3397 (1000 mg/kg of rodent chow for 7 days). No FJ^+^ cells could be found in mice that underwent sham surgery. Bar indicates 90 μm. ***K***, Summarized data for **J**. n = 4 mice per group; * *P* < 0.05 and *** *P* < 0.001 when compared by 1-way ANOVA followed by Tukey’s multiple comparisons test.

We first asked whether the depletion of microglia from the mouse brain would have an effect on the density of neurons in the ischemic cortex. When control mice were fed with rodent diet containing the CSF1R inhibitor PLX3397 (1000 mg/kg of rodent chow) for 3 or 7 days, microglia in their cortex, striatum, and hippocampus were mostly ablated, compared to mice fed with control diet lacking PLX3397 (n = 8 mice per group; on day 3, *P* < 0.0001 for cortex, striatum, and hippocampus; on day 7, *P* < 0.0001 for cortex, striatum, and hippocampus, when compared to mice fed with control diet, Tukey’s multiple comparisons test) (Fig 1A–1D). Moreover, when control mice were fed with the PLX3397-containing diet for 21 days, microglia could not be found in the mouse cortex, striatum, or hippocampus (*P* < 0.0001 for cortex, striatum, and hippocampus, when compared to mice fed with control diet, Tukey’s multiple comparisons test) (Fig 1A–1D). Consistent with a potential role of microglia in regulating the density of neurons after stroke, ischemic stroke caused by dMCAo in mice increased the density of microglia in the ischemic cortex (n = 4 mice per group; *P* < 0.0001 compared to mice that received sham surgery, Tukey’s multiple comparisons test) and resulted in substantial overlap between neurons and microglia (*P* = 0.0024 compared to mice that received sham surgery, Tukey’s multiple comparisons test) (Fig 1E–1G). In comparison, little or no overlap between neurons and microglia could be observed in mice with intact microglia subjected to sham surgery or in mice whose microglia were ablated by PLX3397-containing diet prior to dMCAo (*P* = 0.9909 and *P* = 0.0020 when compared to mice with intact microglia subjected to sham surgery and dMCAo, respectively, Tukey’s multiple comparisons test) (Fig 1E–1G). Importantly, while dMCAo decreased the density of neurons in the ischemic cortex of mice fed with the control diet (*P* < 0.0001 when compared to mice that received sham surgery, Tukey’s multiple comparisons test), ablation of microglia by feeding mice with PLX3397-containing diet prior to dMCAo partially reversed this effect (*P* = 0.0378, compared to mice fed with control diet prior to dMCAo, Tukey’s multiple comparisons test) (Fig 1H). In contrast to the effects on neuronal density in the ischemic cortex, neither dMCAo nor microglial ablation by PLX3397 had an effect on neuronal density in the contralateral non-ischemic cortex (Fig 1I), suggesting that microglia did not regulate the density of healthy non-stressed neurons. To confirm that microglia indeed targeted degenerating neurons, we stained the mouse brain with Fluoro-Jade B (FJ), which selectively stains degenerating neurons but not healthy neurons or other cell types in the brain. Indeed, ischemic stroke caused by dMCAO in mice resulted in a large number of degenerating (FJ^+^) neurons in the ischemic cortex (*P* < 0.0001, compared to mice that received sham surgery, Tukey’s multiple comparisons test), and ablation of microglia by feeding mice with PLX3397-containing diet prior to dMCAo further increased the density of degenerating neurons in this brain region (*P* < 0.0001 and *P* = 0.0101 compared to mice with intact microglia subjected to sham surgery and dMCAo, respectively, Tukey’s multiple comparisons test) (Fig 1I and 1J). Altogether, our data indicate that stroke is associated with increased neuron-to-microglial overlap, and that the presence of microglia decreased the number of degenerating neurons after stroke.

### LPS decreased the density of neurons in the ischemic cortical region in a microglia-dependent manner

**Fig 2:**
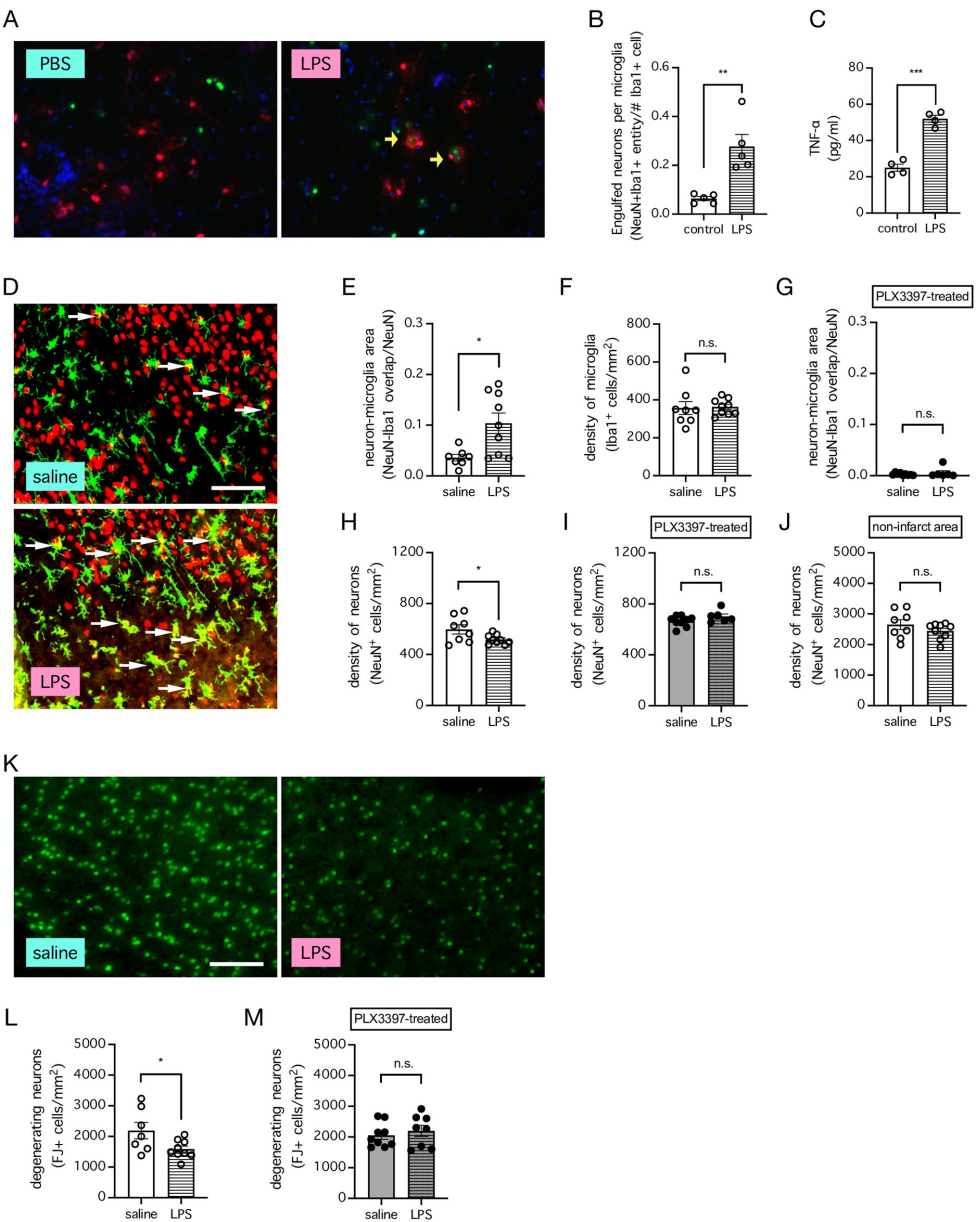
Lipopolysaccharide (LPS) treatment decreases the density of neurons in the ischemic cortical region after stroke in a microglia-dependent manner. ***A***, Representative images showing increased engulfment of neurons (NeuN^+^ cells; *green*) by microglia (Iba1+ cells; *red*) following LPS treatment (0.1 μg/ml) in primary mouse cortical cultures, compared to control treatment with PBS. ***B***, Summarized data for **A**. n = 5 cultures per group. ** *P* < 0.01 when compared by unpaired t test. ***C***, Concentrations of TNF-ɑ in a subset of cultures from **B**. n = 4 cultures per group. *** *P* < 0.001 when compared by unpaired t test. ***D***, Representative images showing neurons (NeuN^+^ cells; *red*) and microglia (Iba1^+^ cells; *green*) in the ischemic cortex of mice subjected to stroke induced by distal middle cerebral arterial occlusion (dMCAo) and were injected with either saline or LPS (0.2 mg/kg, i.p.) 48 h prior to stroke onset. Bar indicates 90 μm. ***E-I***, Summarized data for **D**, showing overlap between NeuN and Iba1 in mice whose microglia were intact (**E**) or depleted by PLX3397 treatment (1000 mg/kg of rodent chow for 7 days) (**G**), the density of microglia (Iba1^+^ cells) in mice whose microglia were intact (**F**), and the density of neurons (NeuN^+^ cells) in mice whose microglia were intact (**H**) or depleted by PLX3397 treatment (1000 mg/kg of rodent chow for 7 days) (**I**), in the ischemic area of the cortex. n = 6–9 mice per group. * *P* < 0.05 when compared by unpaired t test. n.s. indicates no significant difference. ***J***, Same as **H**, but in the contralateral area of the cortex. n = 8–9 mice per group. n.s. indicates no significant difference when compared by unpaired t test. ***K***, Representative images showing degenerating neurons (Fluoro-Jade B^+^ (FJ^+^) cells) in the ischemic cortex of mice subjected to stroke induced by dMCAo and were injected with either saline or LPS (0.2 mg/kg, i.p.) 48 h prior to stroke onset. Bar indicates 90 μm. ***L*,*M***, Summarized data for **K**, showing the density of degenerating neurons (FJ^+^ cells) in the ischemic cortex of mice whose microglia were intact (**L**) or depleted by PLX3397 treatment (1000 mg/kg of rodent chow for 7 days) (**M**). n = 7–9 mice per group. * *P* < 0.05 when compared by unpaired t test. n.s. indicates no significant difference.

Given that LPS and other inflammatory stimuli have been shown to stimulate microglial activities, including phagocytosis, in primary rat cultures [4, 11–14], we next determined the effect of LPS treatment on the density of neurons in a mouse model of stroke, and question whether its effect would be contingent on the presence of microglia. To confirm that LPS used in our experiments can augment microglial activities, we treated primary mouse cortical cultures with either LPS (0.1 μg/ml) or PBS (as a negative control), and found that LPS treatment substantially increased microglial engulfment of neurons, compared to PBS treatment (n = 5 cultures per group; *P* = 0.0027, unpaired t test) (Fig 2A and 2B). Furthermore, LPS treatment was associated with higher concentrations of TNF-ɑ, compared to PBS treatment (n = 4 cultures per group; *P* = 0.0002, unpaired t test) (Fig 2C). In mice subjected to stroke induced by dMCAo, LPS injection (0.2 mg/kg, i.p.), administered 48 h prior to ischemic onset, strongly increased the overlap between neurons (NeuN^+^) and microglia (Iba1^+^), compared to saline injection (n = 7–9 mice per group; *P* = 0.0125, unpaired t test) (Fig 2D and 2E). The increase in the overlap between neurons and microglia was not due to an increase in microglial number, because LPS injection did not have an effect on the number of microglia compared to saline injection (n = 8–9 mice per group; *P* = 0.8657, unpaired t test) (Fig 2F). In addition, the overlap between neurons and microglia could not be an experimental artifact caused by non-specific immuno-staining of neurons or the expression of the microglial marker Iba1 by neurons, because ablation of microglia by PLX3397 prior to stroke completely removed any sign of overlap between neurons and microglia (n = 6–9 mice per group; *P* = 0.4281, unpaired t test) (Fig 2G). Importantly, LPS injection decreased the density of neurons (NeuN^+^ cells) in the ischemic cortex of mice after stroke, compared to saline injection (n = 8–9 mice per group; *P* = 0.0435, unpaired t test) (Fig 2H), and this effect was completely abolished when microglia were ablated by PLX3397 treatment prior to stroke (n = 6–9 mice per group; *P* = 0.1340, unpaired t test) (Fig 2I) and not seen in the contralateral cortex of the mice with intact microglia (n = 8–9 mice per group; *P* = 0.2629, unpaired t test) (Fig 2J). Therefore, LPS, which can stimulate microglia activities, can decrease the number of neurons in the ischemic cortex in a microglia-dependent manner but has no effect on neurons in the contralateral non-ischemic cortex. To determine whether the effect of LPS injection applies to degenerating neurons, we stained the mouse brain with FJ and found that LPS decreased the number of degenerating neurons (FJ^+^ cells) after stroke in mice with intact microglia (n = 7–9 mice per group; *P* = 0.352, unpaired t test) (Fig 2K and 2L) but had no effect in mice whose microglia were ablated by PLX3397 treatment prior to stroke (n = 8–9 mice per group; *P* = 0.5238, unpaired t test) (Fig 2M). These data together showed that LPS, in a microglia-dependent manner, can augment neuronal loss after stroke, and that the effect is somewhat specific to the degenerating neurons in the ischemic region but not the healthy neurons in non-ischemic regions.

### Inhibitor of MFG-E8 signaling increased the density of neurons in the ischemic cortical region in a microglia-dependent manner

**Fig 3:**
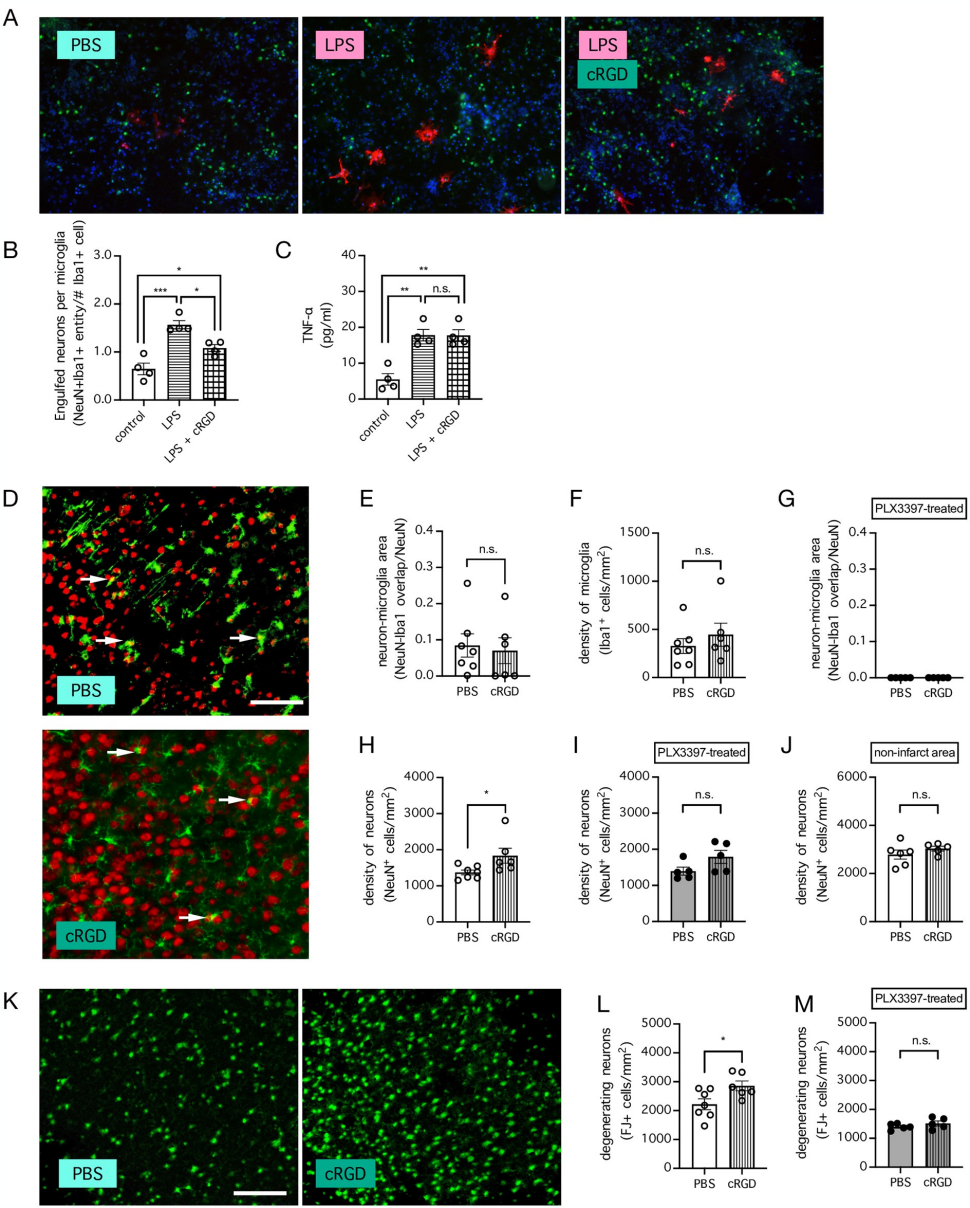
cRGD treatment increases the density of neurons in the ischemic cortical region after stroke. ***A***, Representative images showing increased engulfment of neurons (NeuN^+^ cells; *green*) by microglia (Iba1+ cells; *red*) following LPS treatment (0.1 μg/ml) in primary mouse cortical cultures, compared to control treatment with PBS, and its inhibition in the presence of cRGD (50 μM). ***B***, Summarized data for **A**. n = 4 cultures per group. * *P* < 0.05 and *** *P* < 0.001 when compared by 1-way ANOVA followed by Tukey’s multiple comparisons test. ***C***, Concentrations of TNF-ɑ in a subset of cultures from **B**. n = 4 cultures per group. ** *P* < 0.01 when compared by 1-way ANOVA followed by Tukey’s multiple comparisons test. n.s. indicates no significant difference. ***D***, Representative images showing neurons (NeuN^+^ cells; *red*) and microglia (Iba1^+^ cells; *green*) in the ischemic cortex of mice subjected to stroke induced by distal middle cerebral arterial occlusion (dMCAo) and were injected with either PBS or cRGD (1 mM x 5 μl, i.c.v. into the contralateral hemisphere) immediately prior to ischemic onset. Bar indicates 90 μm. ***E-I***, Summarized data for **D**, showing overlap between NeuN and Iba1 in mice whose microglia were intact (**E**) or depleted by PLX3397 treatment (1000 mg/kg of rodent chow for 7 days) (**G**), the density of microglia (Iba1^+^ cells) in mice whose microglia were intact (**F**), and the density of neurons (NeuN^+^ cells) in mice whose microglia were intact (**H**) or depleted by PLX3397 treatment (1000 mg/kg of rodent chow for 7 days) (**I**), in the ischemic area of the cortex. For mice fed with control diet, n = 6–7 mice per group, and for mice fed with PLX3397-containing diet, n = 5 mice per group. * *P* < 0.05 when compared by unpaired t test. n.s. indicates no significant difference. ***J***, Same as **H**, but in the contralateral area of the cortex. n = 5–6 mice per group. n.s. indicates no significant difference when compared by unpaired t test. ***K***, Representative images showing degenerating neurons (Fluoro-Jade B^+^ (FJ^+^) cells) in the ischemic cortex of mice subjected to stroke induced by dMCAo and were injected with either PBS or cRGD (1 mM x 5 μl, i.c.v. into the contralateral hemisphere) immediately prior to ischemic onset. Bar indicates 90 μm. ***L*,*M***, Summarized data for **K**, showing the density of degenerating neurons (FJ^+^ cells) in the ischemic cortex of mice whose microglia were intact (**L**) or depleted by PLX3397 treatment (1000 mg/kg of rodent chow for 7 days) (**M**). For mice fed with control diet, n = 6–7 mice per group, and for mice fed with PLX3397-containing diet, n = 5 mice per group. * *P* < 0.05 when compared by unpaired t test. n.s. indicates no significant difference.

MFG-E8 signaling has been shown to be essential for microglial phagocytosis *in vitro* [4, 11–14], and the cyclic peptide cRGD, which mimics the RGD (arginine-glycine-aspartate) domain of MFG-E8, has been shown to prevent MFG-E8 binding to ɑ_v_β_3_ integrin-expressing cells, thereby inhibiting MFG-E8-mediated phagocytosis [38]. Consistent with the reported role of MFG-E8 signaling in phagocytosis, cRGD (50 μM) inhibited LPS-induced microglial engulfment of neurons in our primary cortical cultures (n = 4 cultures per group; *P* = 0.0002 for LPS compared to control and *P* = 0.0149 for LPS in the presence of cRGD compared to LPS alone, Tukey’s multiple comparisons test) (Fig 3A and 3B). In contrast to its effect on microglial phagocytosis, cRGD had no effect on LPS-induced release of TNF-ɑ (n = 4 cultures per group; *P* = 0.0011 for LPS compared to control and *P* = 0.9995 for LPS in the presence of cRGD compared to LPS alone, Tukey’s multiple comparisons test) (Fig 3C), suggesting that the inhibitory effect of cRGD on microglia is somewhat specific to phagocytosis. In the mouse model of stoke, compared to PBS treatment, cRGD treatment (1 mM x 5 μl, i.c.v. into the contralateral hemisphere) immediately prior to ischemic onset had no effect on the interaction between neurons (NeuN^+^) and microglia (Iba1^+^) (n = 6–7 mice per group; *P* = 0.7686, unpaired t test) (Fig 3D and 3E) or the density of microglia (*P* = 0.4122, unpaired t test) (Fig 3F) in mice subjected to stroke induced by dMCAo. Nevertheless, cRGD treatment increased the density of neurons (NeuN^+^ cells) in the ischemic cortex of these mice (*P* = 0.0440, unpaired t test) (Fig 3H), indicating that MFG-E8 signaling contributed to neuronal loss in the ischemic infarct after stroke. The inhibitory effect of cRGD treatment on neuronal loss after stroke could not be explained by non-specific inhibition of phagocytosis by other cell types, such as astrocytes, because it was abolished when microglia were depleted by PLX3397 treatment prior to ischemic onset (n = 5 mice per group; *P* = 0.0960, unpaired t test) (Fig 3G and 3I). In addition, cRGD treatment had no effect on the density of neurons in the contralateral non-ischemic cortex (n = 5–6 mice per group; *P* = 0.3048, unpaired t test) (Fig 3J), suggesting that MFG-E8 signaling after stroke has no effect on the density of healthy neurons. To confirm that degenerating neurons are targets of MFG-E8 signaling after stroke, we stained the mouse brain with FJ and found that cRGD treatment further increased the number of degenerating neurons (FJ^+^ cells) after stroke in mice with intact microglia (n = 6–7 mice per group; *P* = 0.0299, unpaired t test) (Fig 3K and 3L) but had no effect in mice whose microglia were depleted by PLX3397 treatment prior to stroke (n = 5 mice per group; *P* = 0.2771, unpaired t test) (Fig 3M). Notably, the effect of cRGD treatment on the number of degenerating neurons could not be due to inadvertent injury caused by the i.c.v. injection procedure, because all injections were made only to the contralateral non-ischemic hemisphere. Therefore, these data further demonstrated that MFG-E8 signaling targeted degenerating neurons after stroke in a microglia-dependent manner.

### MFG-E8 signaling protects the brain from ischemic infarction after stroke in a microglia-dependent manner

**Fig 4:**
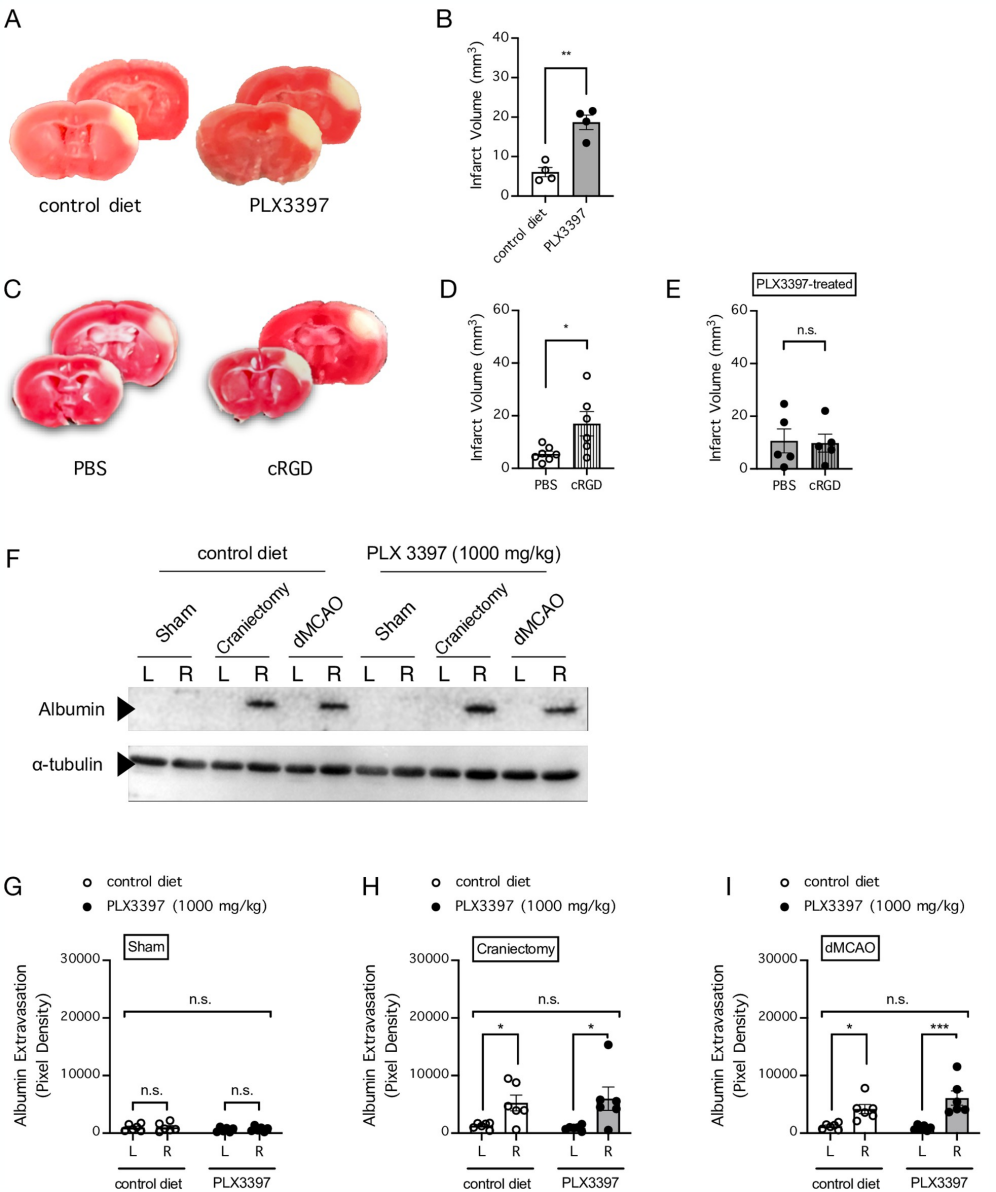
cRGD treatment or microglial depletion augments the development of cerebral infarction and has no effect on blood-brain barrier (BBB) permeability after stroke. ***A***, Representative images showing triphenyltetrazolium chloride (TTC)-stained coronal brain sections from mice whose microglia were intact (control diet) or depleted by PLX3397 treatment (1000 mg/kg of rodent chow for 7 days) and were subjected to stroke induced by distal middle cerebral arterial occlusion (dMCAo). ***B***, Summarized data for **A**. n = 4 mice per group. ** *P* < 0.01 when compared by unpaired t test. ***C***, Representative images showing TTC-stained coronal brain sections from mice that received PBS or cRGD treatment (1 mM x 5 μl, i.c.v. into the contralateral hemisphere) and were subjected to stroke induced by dMCAo. ***D***, Summarized data for **C**. n = 6–7 mice per group. * *P* < 0.05 when compared by unpaired t test. ***E***, Same as **D**, but microglia were depleted by PLX3397 treatment (1000 mg/kg of rodent chow for 7 days) prior to ischemia. n = 5 mice per group. n.s. indicates no significant difference when compared by unpaired t test. ***F***, Representative images of western blots showing extravasation of circulating endogenous albumin into cerebral parenchyma of the right (ipsilateral) but not left (contralateral) hemisphere in mice whose microglia were intact or depleted by PLX3397 treatment (1000 mg/kg of rodent chow for 7 days) prior to ischemia and subjected to sham surgery or BBB disruption caused by craniectomy or dMCAo. ***G-I***, Summarized data for F, showing albumin extravasation in mice subjected to sham surgery (**G**), craniectomy (**H**), or dMCAo (**I**). n = 6 mice per group. * *P* < 0.05 and *** *P* < 0.001 when compared by 2-way repeated-measures ANOVA, in which the right and left hemispheres from the same mice were matched, followed by Holm-Sidak’s multiple comparisons test. n.s. indicates no significant difference.

Development of cerebral infarction is a hallmark of stroke pathogenesis. In mice subjected to dMCAo, the development of cerebral infarction is a gradual process that expands from a smaller cortical area 4 mm from lambda at 3–6 h post-ischemia, and reaches a plateau by 24 h post-ischemia, at which point the final infarct volume can span a range from 1 mm to 5 mm from lambda [39]. To determine whether microglial MFG-E8 signaling has an effect on the development of cerebral infarction after stroke induced by dMCAo, we first compared the cerebral infarction in mice whose microglia were intact or depleted by PLX3397 treatment (Fig 4A and 4B). Interestingly, mice whose microglia were depleted developed a substantially larger cerebral infarction after stroke, compared to mice whose microglia were intact (n = 4 mice per group; *P* = 0.0012, unpaired t test) (Fig 4A and 4B). Likewise, mice treated with cRGD (1 mM x 5 μl, i.c.v. into the contralateral hemisphere) developed a substantially larger cerebral infarction after stroke, compared to mice treated with PBS (n = 6–7 mice per group; *P* = 0.0246, unpaired t test) (Fig 4C and 4D). These data are consistent with the notion that microglial MFG-E8 signaling protected the mouse brain from the development of cerebral infarction after stroke. Importantly, the effect of cRGD treatment on the development of cerebral infarction was completely abolished in mice whose microglia were depleted by PLX3397 treatment (n = 5 mice per group; *P* = 0.8864, unpaired t test) (Fig 4E).

### No role of microglia in blood-brain barrier (BBB) disruption after stroke

The severity of neuronal injury or cortical infarction often correlates with the degree of BBB disruption after stroke [40–44], and drugs that are neuroprotective against neuronal injury could protect against BBB disruption in animal stroke models [45, 46]. Given the effect that the presence of microglia has on neuronal density and cortical infarction, we asked whether microglia would have a protective or disruptive role in BBB disruption in our stroke model. Consistent with reported findings [37], craniectomy and dMCAo but not sham treatment induced prominent unilateral BBB disruption, characterized by extravasation of endogenous serum albumin into the ipsilateral (right) cortex and not the contralateral (left) cortex (n = 6 mice per group; for sham treatment, *P* = 0.8309 for mice fed with control diet and *P* = 0.6551 for mice fed with PLX3397-containing diet; for the craniectomy procedure, *P* = 0.0372 for mice fed with control diet and *P* = 0.0230 for mice fed with PLX3397-containing diet; for stroke induced by dMCAo, *P* = 0.0124 for mice fed with control diet and *P* = 0.0007 for mice fed with PLX3397-containing diet, Holm-Sidak’s multiple comparisons test) (Fig 4F–4I). However, depletion of microglia in mice fed with the PLX3397-containing diet had no effect on the basal level of BBB permeability or on the magnitude of BBB disruption, compared to mice fed with control diet (*P* = 0.2885 for mice with sham treatment, *P* = 0.8943 for mice with the craniectomy procedure, and *P* = 0.3360 for mice with stroke induced by dMCAo, 2-way repeated-measures ANOVA matching data from the right and left cortices of the same mouse) (Fig 4F–4I). Therefore, despite the important roles of microglia in neuronal loss and the development of cortical infarction, our data showed that microglia are not involved in BBB disruption after stroke induced by dMCAo.

## Discussion

Microglia and other tissue-resident macrophages are known to phagocytose viable neurons and apoptotic cells through their interactions via the RGD (arginine-glycine-aspartate) domain of the extracellular MFG-E8 protein [13, 38]. In the case of zebrafish microglia, once the targeted cells are phagocytosed, the phagosomes fuse with lysosomes to enable degradation and recycling of cellular components [47]. In this study, we further showed that microglial MFG-E8 signaling contributed significantly to neuronal loss in the ischemic cortex in a mouse model of stroke, but had no effect on the density of neurons in non-ischemic brain regions. Therefore, in our stroke model, this microglia-mediated neuronal loss was an active process during cerebral ischemia, but remained dormant in otherwise healthy brain tissue. Notably, our data are generally consistent with other studies in which microglial phagocytosis triggered by inflammatory stimuli can contribute to neuronal loss in primary neuronal cultures *in vitro* [4, 11–14]; nevertheless, in those studies, perhaps because neuronal loss is generally considered a pathogenic process in neurodegenerative diseases, the authors have all concluded based on such data that microglial phagocytosis could be a causative mechanism in the pathogenesis of neurodegenerative diseases. Contrary to their conclusion, we further demonstrated in this study that microglial MFG-E8 signaling, which is required for phagocytosis of neurons, while indeed contributing partially to neuronal loss after stroke, is a protective measure to minimize the development of cerebral infarction.

The discovery of CSF1R inhibitors, including PLX3397, as simple and effective pharmacological tools to deplete microglia and thereby verify their functions has made it possible to confirm experimental findings regarding microglia and reconcile existing contradictions [48]. For example, in light of contradictory reports claiming that microglia can promote cocaine addictive behaviors through the release of IL-1β [49] and inhibit these behaviors through the release of TNF-ɑ [50], it has recently been reported that mice whose microglia were depleted by PLX3397 treatment had similar behavioral responses and sensitization to cocaine compared to mice with intact microglia [31]. In the present study, we exploited this strategy to confirm that the observed effects of LPS treatment, which was used to trigger neuroinflammation, and cRGD treatment, which was used to inhibit MFG-E8 signaling, were microglia-dependent. Indeed, in mice whose microglia were depleted prior to the experiment, LPS and cRGD treatments had no effect on microglial engulfment of neurons *in vitro*, the density of neurons in the ischemic brain region *in vivo*, or the development of cortical infarction. This was especially important given that other cell types in the brain, such as astrocytes, also exhibit important phagocytic activities and can have phagocytic efficiency and priorities different than those of microglia [3, 21–23]. Notably, contrary to our findings, numerous studies have previously found that global disruption of phagocytosis in a non-cell-type-specific manner can minimize disease progression in animal models of neurodegenerative diseases [13–16]. Further investigations based on cell-type-specific depletion could be able to confirm whether phagocytosis by microglia, astrocytes, or other cell types contributed to the disease progression observed in those studies.

Several early studies have suggested that microglia are a major causative factor in BBB disruption after stroke. For example, microglia tend to extend their cellular protrusions toward blood vessels after stroke, and they are found to contain tight-junction proteins normally expressed by endothelial cells that maintain the BBB, suggesting that microglia could cause BBB disruption by phagocytosing endothelial cells [51]. In addition, minocycline, which inhibits hypoxic microglial activation *in vitro*, protects astrocytes and endothelial cells against hypoxic cell death *in vitro* and protects the brain against BBB disruption after stroke *in vivo* [52], and mice with genetic deletion of Cx3Cr1, which is expressed by microglia and not neurons and astrocytes in the brain parenchyma, have altered microglial behavior and less BBB disruption after stroke [51, 53]. However, it should be noted that minocycline is not microglia-specific and genetic deletion of Cx3Cr1 only alters rather than completely abolishes microglial behavior; therefore, these and other observational data could not unequivocally conclude that microglia cause BBB disruption after stroke. On the other hand, at least one study has previously shown that BBB permeability to dextran and immunoglobulin after stroke was not affected in mice whose microglia were depleted compared to mice with intact microglia [18], raising the intriguing possibility that microglia do not play a role in BBB disruption after stroke. However, we and others have reported evidence to show that dextran is not a suitable indicator of BBB disruption after stroke [37, 54] and that immunoglobulin does not efficiently cross the BBB in the early phase of stroke [54]. In this study, we provide new evidence to confirm that BBB disruption in mice subjected to stroke induced by dMCAo led to substantial unilateral leakage of endogenous serum albumin into the brain and that there was no difference in the severity of BBB disruption between mice with intact microglia and mice whose microglia were depleted prior to stroke. Therefore, at least in our stroke model, microglia do not contribute to BBB disruption after stroke.

There was a number of limitations in our study that warrants future research. (1) Despite the well-established role of MFG-E8 signaling in microglial phagocytosis of neurons [13, 38], we have limited our aim to focus specifically on the role of microglial MFG-E8 signaling in the development of cortical infarction, but have made little attempt to also ask whether phagocytosis in general or phagocytosis of neurons in particular played a role in this process. Therefore, although our data may intrigue the notion that microglial phagocytosis of degenerating neurons can limit the development of cortical infarction, it should be noted that further experiments with concrete evidence for the role phagocytosis in this process will be required to make such a conclusion. (2) In addition to the phagocytosis of neurons, microglial phagocytosis of other cell types that also depends on MFG-E8 signaling could also affect the interpretation of our *in vivo* data. Indeed, microglial phagocytosis of neutrophils was reported to be a neuroprotective mechanism after stroke [55]. (3) Although we and others have demonstrated that ablation of microglia by CSF1R inhibition can increase brain infarction after stroke [18–20], ablation of diphtheria toxin receptor-labelled microglia in genetically engineered mice was found to decrease brain infarction in a mouse model of stroke [56]. The explanation for the discrepancy in these findings due to different methods of microglial ablation is unclear, but raises the intriguing possibility that these widely used methods for removal of microglia can have side effects that are yet unknown and can dramatically alter the outcome of neurodegeneration in these experimental models. (4) Recent advancements in single cell RNA sequencing have led to the discovery of up to 12 different subtypes or transcriptional states of microglia, some of which are either positively or negatively correlated with neurodegenerative diseases [57, 58]. This could explain some of the discrepancies in experimental findings from different studies that explored the role of microglia in general or phagocytosis in particular in animal models of stroke. Future studies could investigate whether different microglial subtypes or transcriptional states are more vulnerable to different methods of microglial ablation or inhibition of MFG-E8 signaling by cRGD. (5) Following recent evidence that demonstrated that craniectomy can cause BBB disruption [37, 59], and dMCAo can further exacerbate that disruption [37], a secondary aim of the study was to determine whether microglial MFG-E8 signaling participated in BBB disruption in either scenarios. However, we made no attempt to also investigate whether microglial MFG-E8 signaling participated in the mechanism of BBB disruption by other procedures, including intracarotid injection of hyperosmolar mannitol, focused ultrasound coupled with intravenous microbubbles, and inhalation of carbogen, or other models of stroke. Therefore, our finding that microglial MFG-E8 does not play a role in BBB disruption cannot to generalized to describe the mechanism of BBB disruption induced by other means.

In conclusion, we confirmed in this study that the presence of microglia is a significant contributor to neuronal loss in ischemic brain infarct. This process of neuronal loss specifically targeted stressed and degenerating neurons in the ischemic brain region and not healthy non-ischemic neurons in other brain regions. Importantly, the neuronal loss was a protective mechanism to prevent the development and spread of cortical infarction after stroke. Given that stroke continues to be a leading cause of death and disability with limited treatment options [60], future studies could consider pharmaceutical developments related to facilitating microglia-mediated clearance of degenerating neurons as a strategy to improve stroke outcome. Furthermore, the findings reported here can be extended to better understand the pathogenesis of other neurodegenerative diseases beyond ischemic stroke.

## Supporting information

S1 File Original western blot images of Fig 4F–4I. Original western blot images showing extravasation of circulating endogenous albumin into cerebral parenchyma of the right (ipsilateral) but not left (contralateral) hemisphere in mice whose microglia were intact or depleted by PLX3397 treatment (1000 mg/kg of rodent chow for 7 days) prior to ischemia and subjected to sham surgery or BBB disruption caused by craniectomy or dMCAo.
(PPTX)
