# Supplementary material for: Microglia through MFG-E8 signaling decrease the density of degenerating neurons and protect the brain from the development of cortical infarction after stroke
Source: PLoS One. 2024 Aug 7;19(8):e0308464. doi: 10.1371/journal.pone.0308464 (PMC11305554; doi:10.1371/journal.pone.0308464)
Supplement: S1 File — Original western blot images showing extravasation of circulating endogenous albumin into cerebral parenchyma of the right (ipsilateral) but not left (contralateral) hemisphere in mice whose microglia were intact or depleted by PLX3397 treatment (1000 mg/kg of rodent chow for 7 days) prior to ischemia and subjected to sham surgery or BBB disruption caused by craniectomy or dMCAo. (PPTX) [file pone.0308464.s001.pptx]

## Slide 1
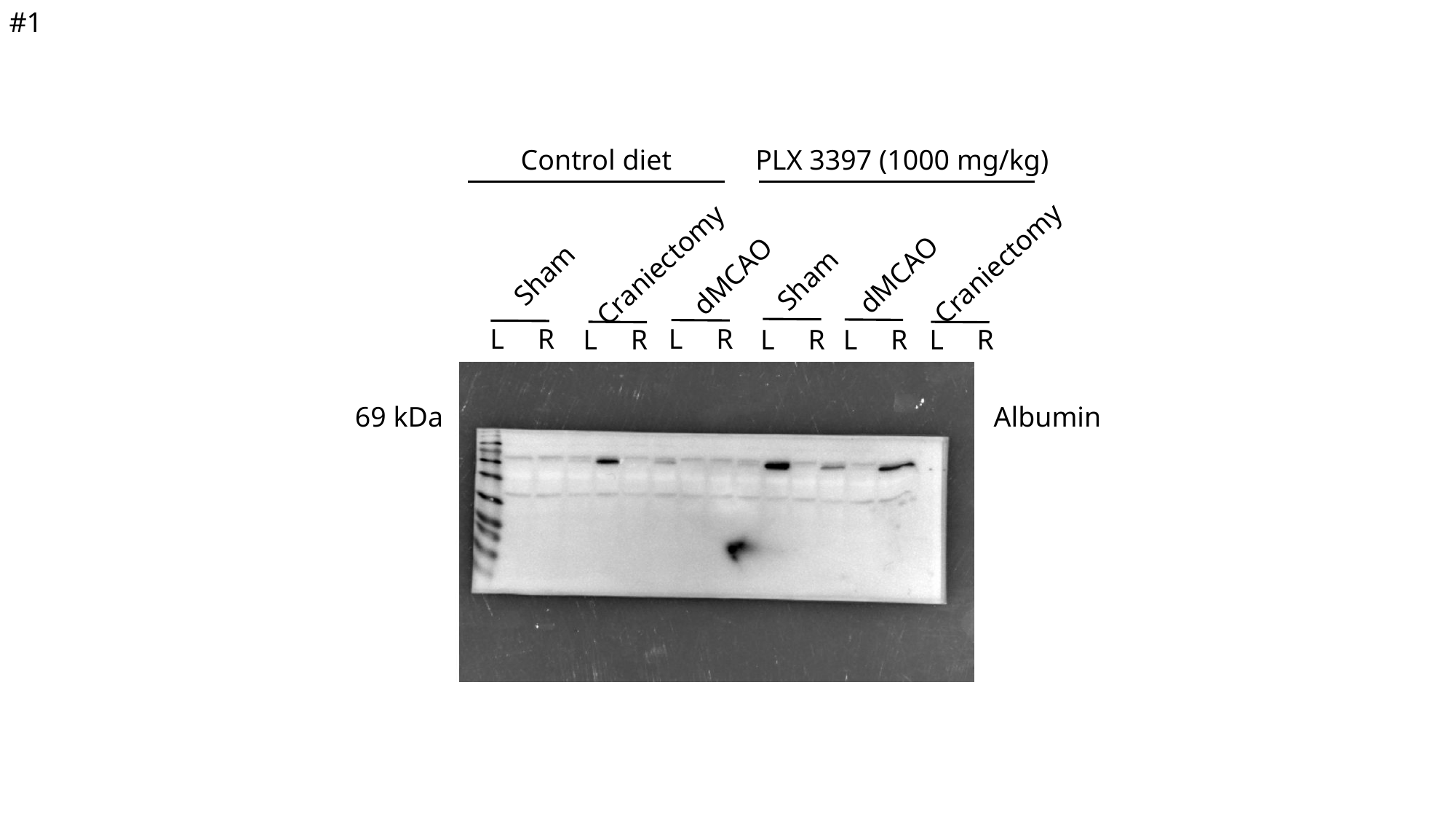

#1
Control diet
PLX 3397 (1000 mg/kg)
Craniectomy
Craniectomy
Sham
dMCAO
dMCAO
Sham
L
R
L
R
L
R
L
R
L
R
L
R
69 kDa
Albumin

## Slide 2
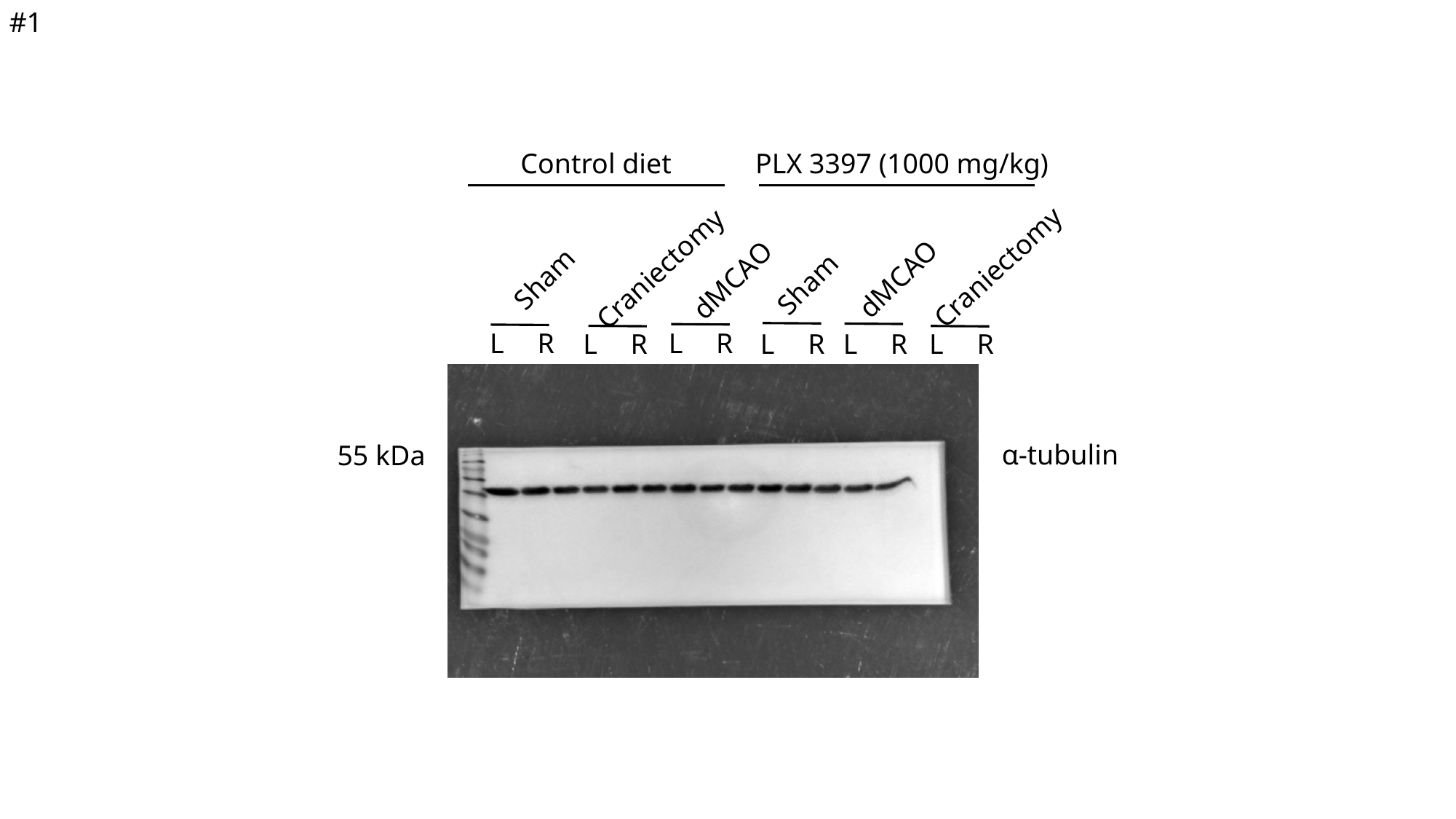

#1
Control diet
PLX 3397 (1000 mg/kg)
Craniectomy
Craniectomy
Sham
dMCAO
dMCAO
Sham
L
R
L
R
L
R
L
R
L
R
L
R
α-tubulin
55 kDa

## Slide 3
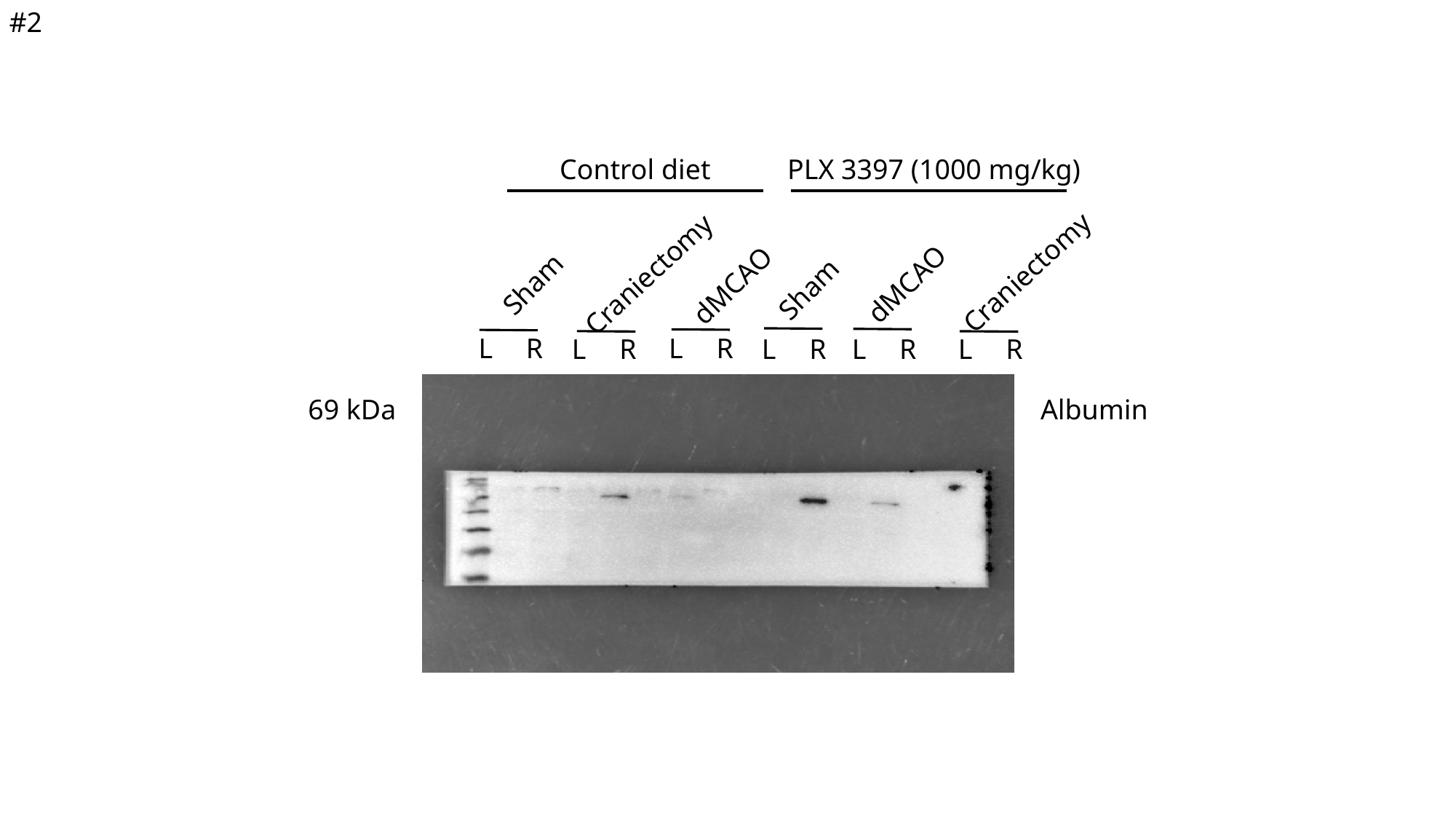

#2
Control diet
PLX 3397 (1000 mg/kg)
Craniectomy
Craniectomy
Sham
dMCAO
dMCAO
Sham
L
R
L
R
L
R
L
R
L
R
L
R
69 kDa
Albumin

## Slide 4
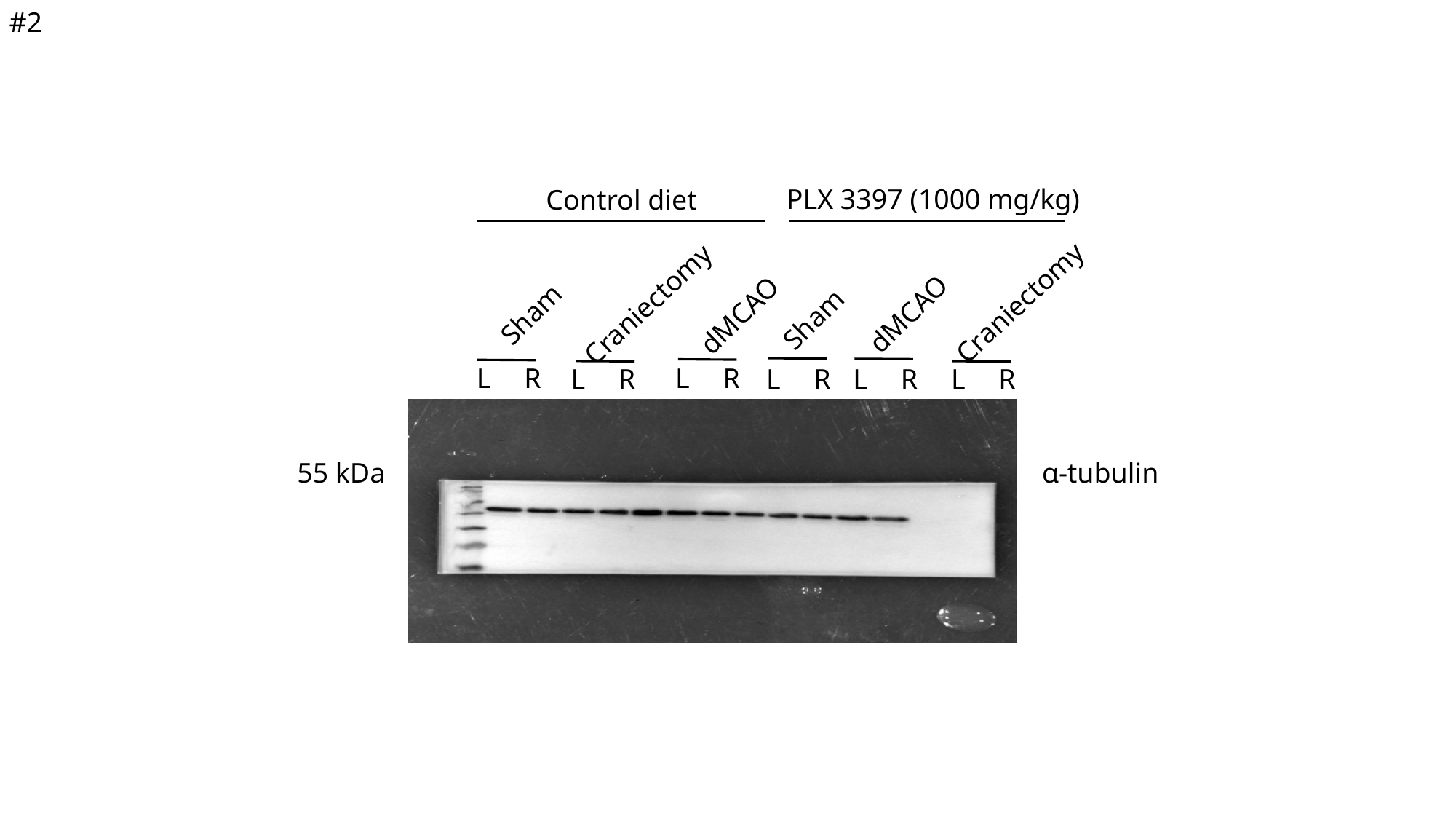

#2
PLX 3397 (1000 mg/kg)
Control diet
Craniectomy
Craniectomy
Sham
dMCAO
dMCAO
Sham
L
R
L
R
L
R
L
R
L
R
L
R
55 kDa
α-tubulin

## Slide 5
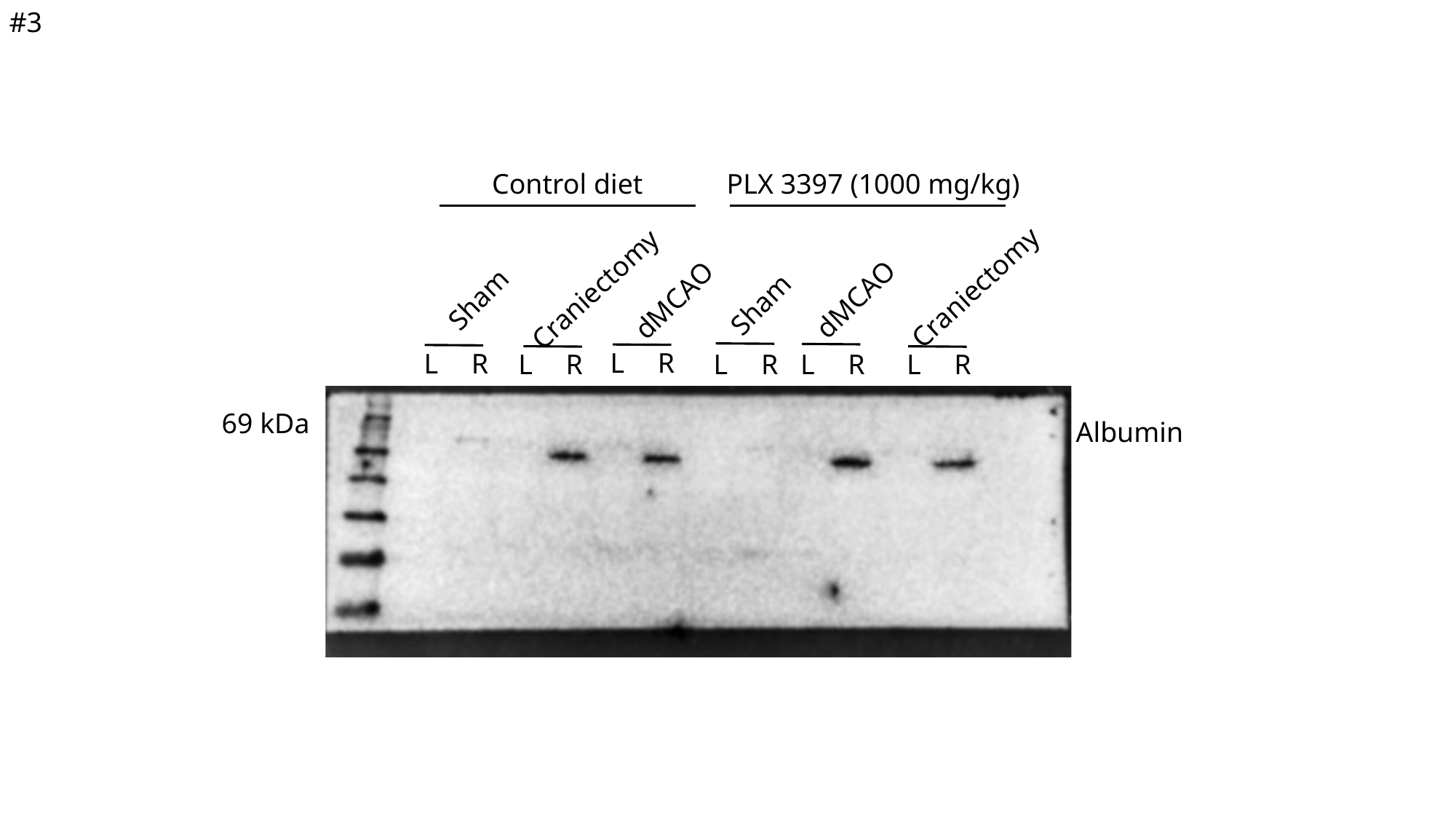

#3
Control diet
PLX 3397 (1000 mg/kg)
Craniectomy
Craniectomy
Sham
dMCAO
dMCAO
Sham
L
R
L
R
L
R
L
R
L
R
L
R
69 kDa
Albumin

## Slide 6
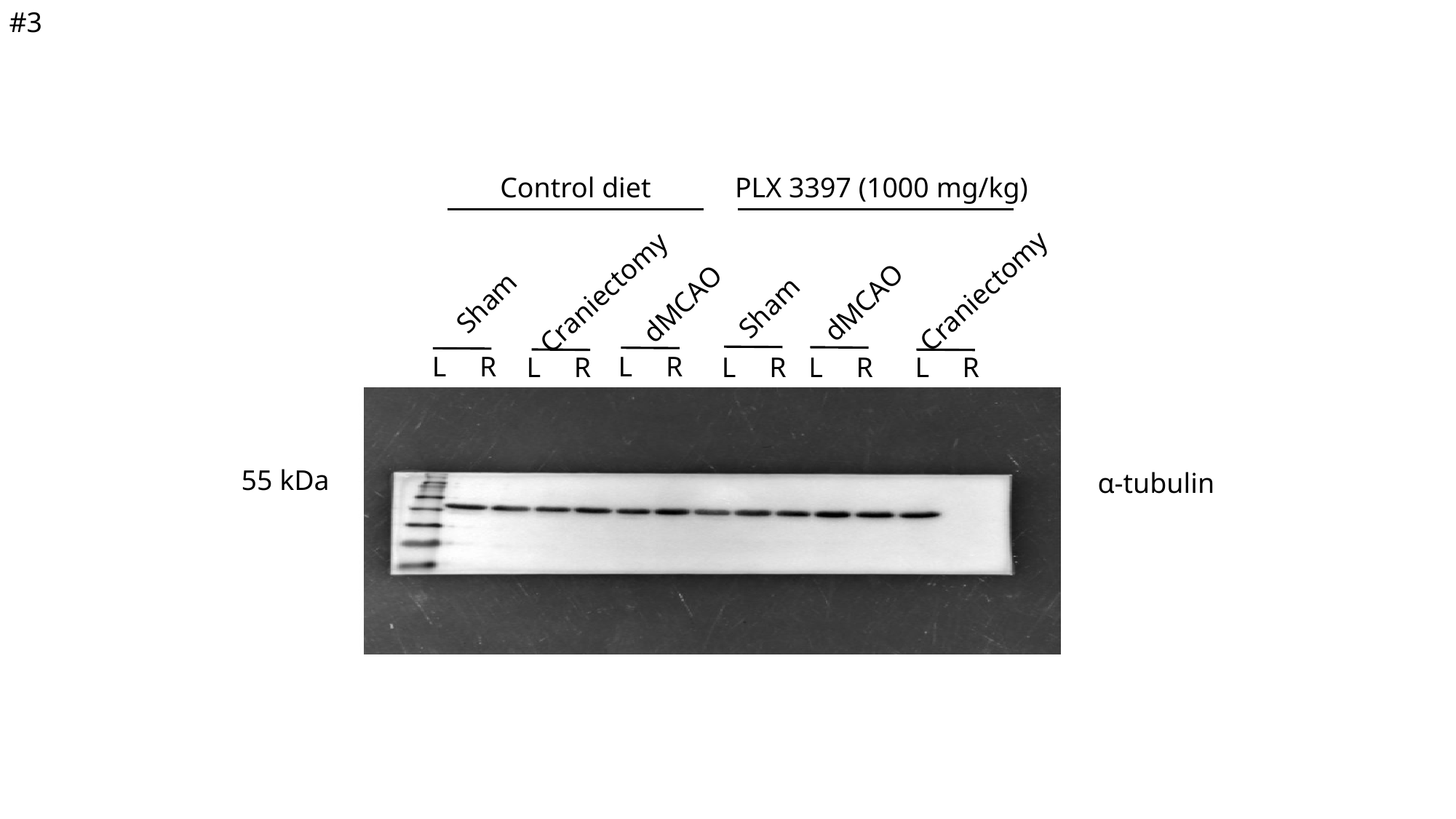

#3
Control diet
PLX 3397 (1000 mg/kg)
Craniectomy
Craniectomy
Sham
dMCAO
dMCAO
Sham
L
R
L
R
L
R
L
R
L
R
L
R
55 kDa
α-tubulin

## Slide 7
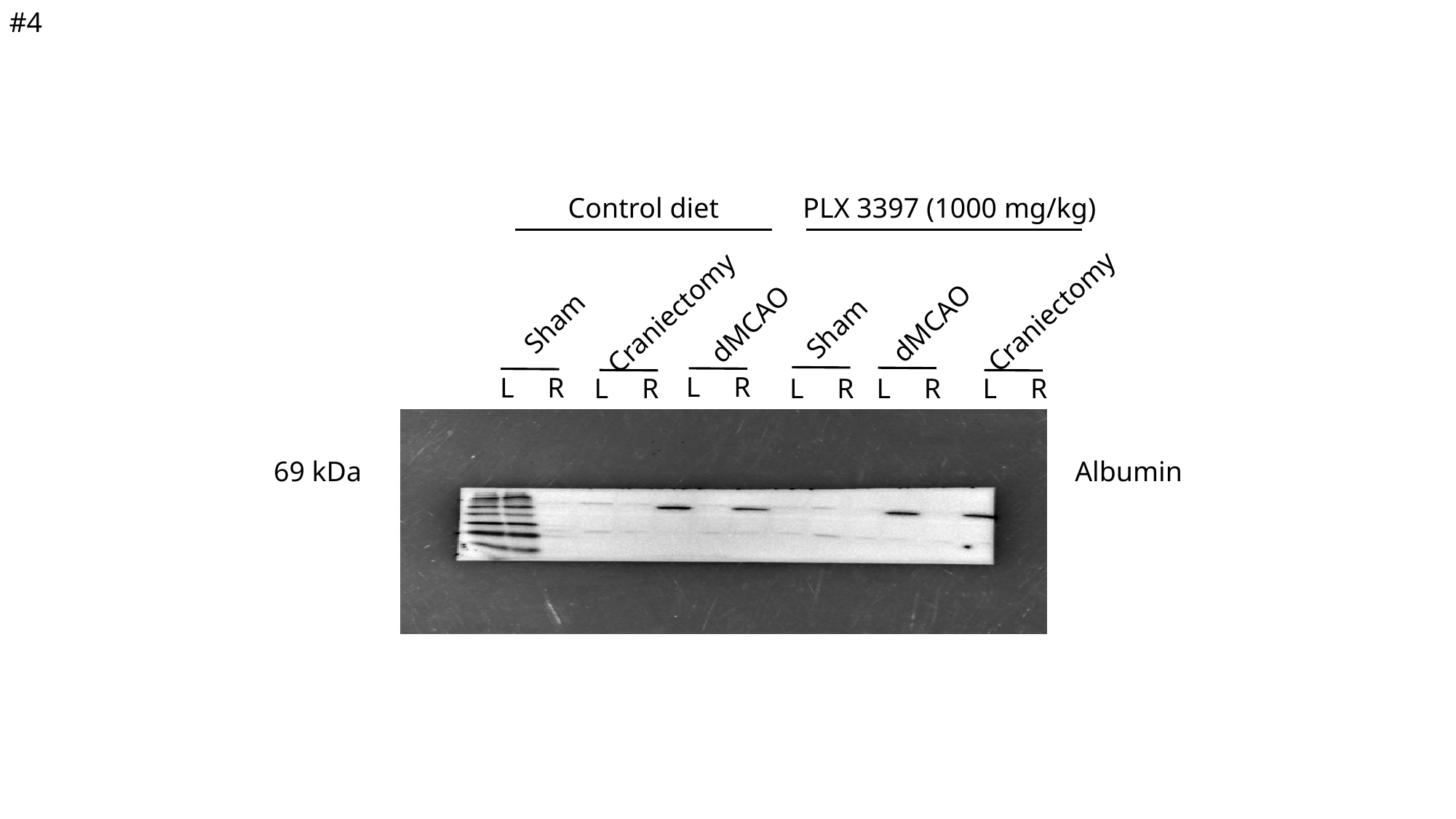

#4
Control diet
PLX 3397 (1000 mg/kg)
Craniectomy
Craniectomy
Sham
dMCAO
dMCAO
Sham
L
R
L
R
L
R
L
R
L
R
L
R
69 kDa
Albumin

## Slide 8
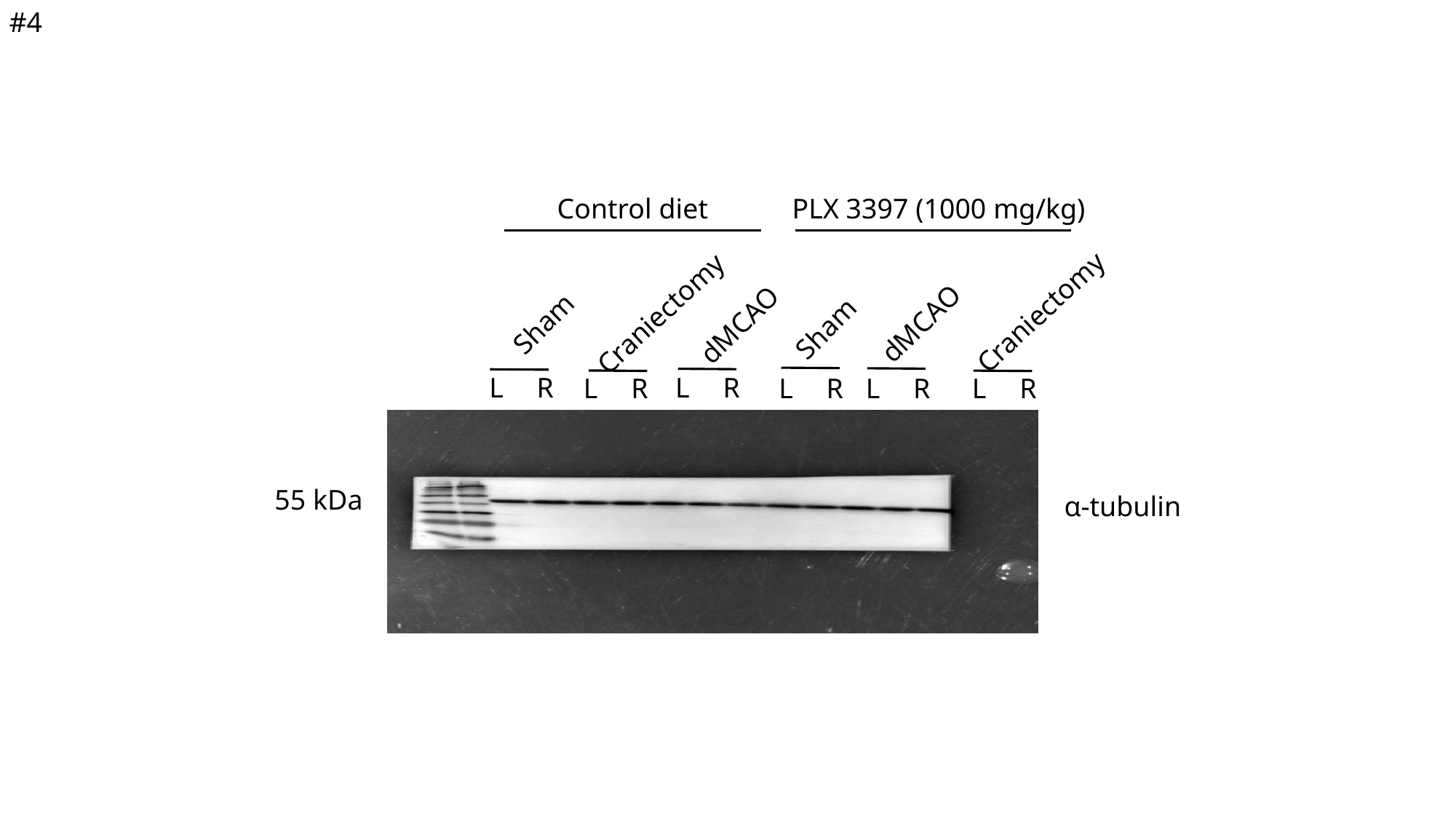

#4
Control diet
PLX 3397 (1000 mg/kg)
Craniectomy
Craniectomy
Sham
dMCAO
dMCAO
Sham
L
R
L
R
L
R
L
R
L
R
L
R
55 kDa
α-tubulin

## Slide 9
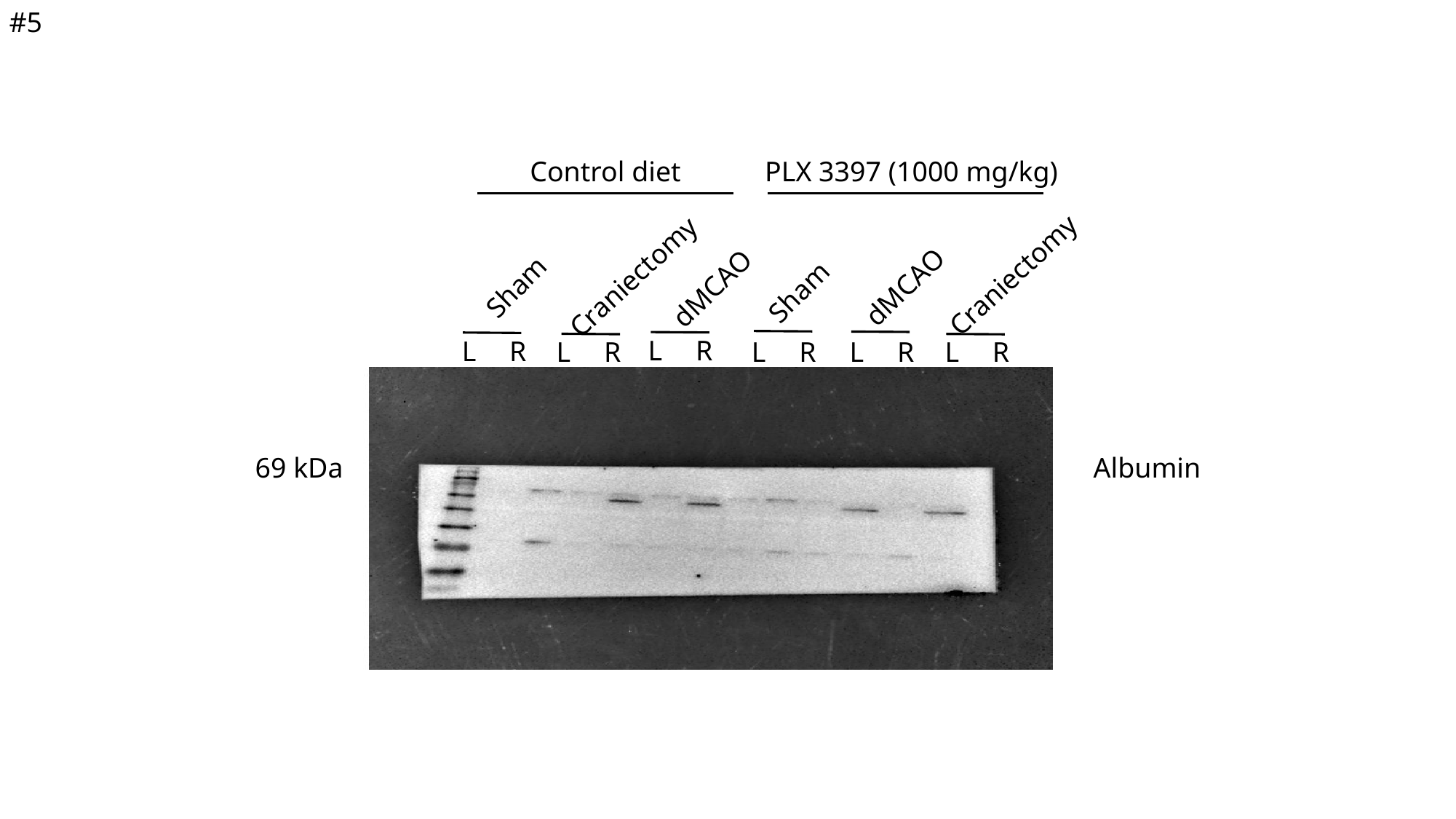

#5
Control diet
PLX 3397 (1000 mg/kg)
Craniectomy
Craniectomy
Sham
dMCAO
dMCAO
Sham
L
R
L
R
L
R
L
R
L
R
L
R
69 kDa
Albumin

## Slide 10
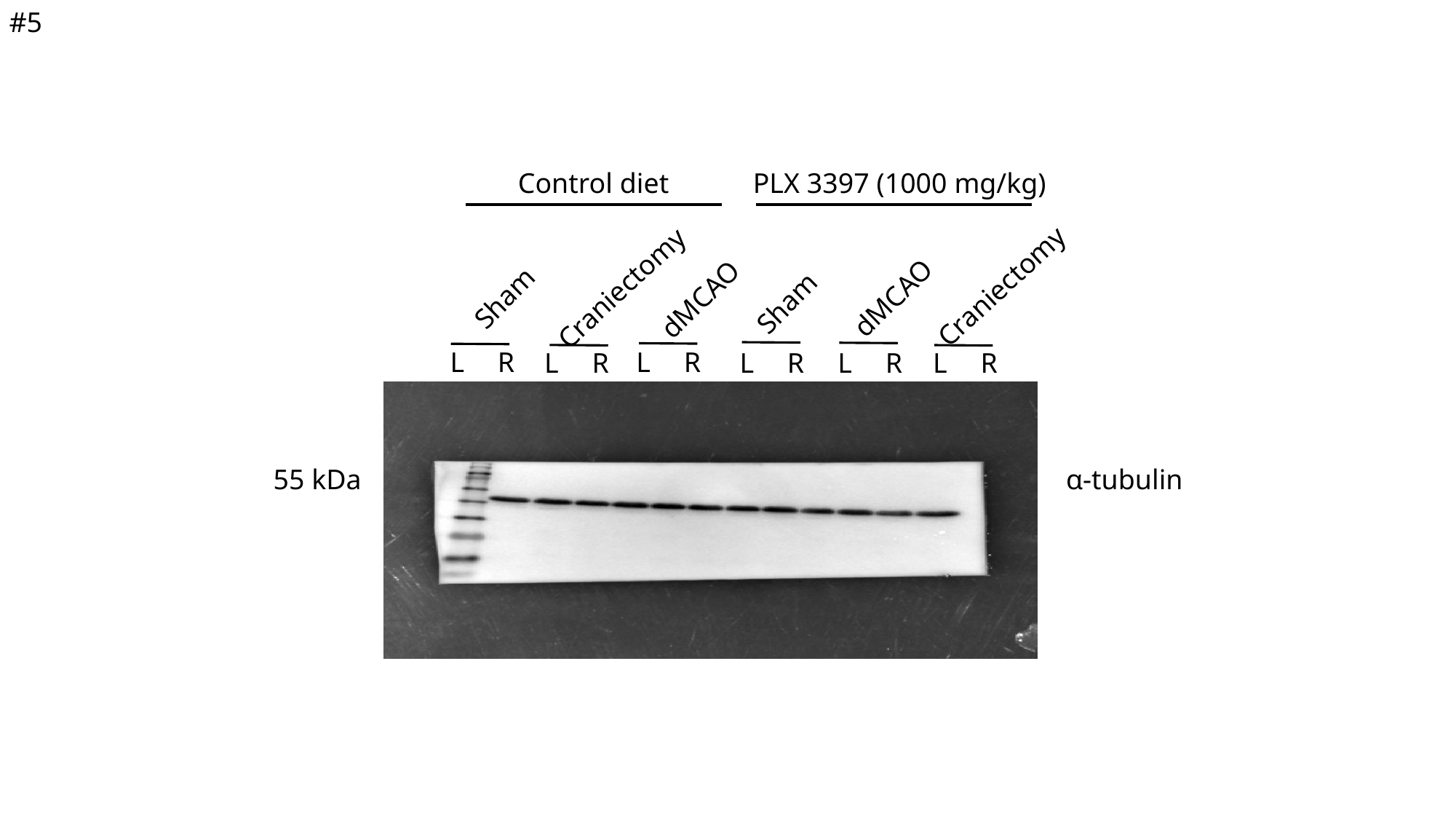

#5
Control diet
PLX 3397 (1000 mg/kg)
Craniectomy
Craniectomy
Sham
dMCAO
dMCAO
Sham
L
R
L
R
L
R
L
R
L
R
L
R
55 kDa
α-tubulin

## Slide 11
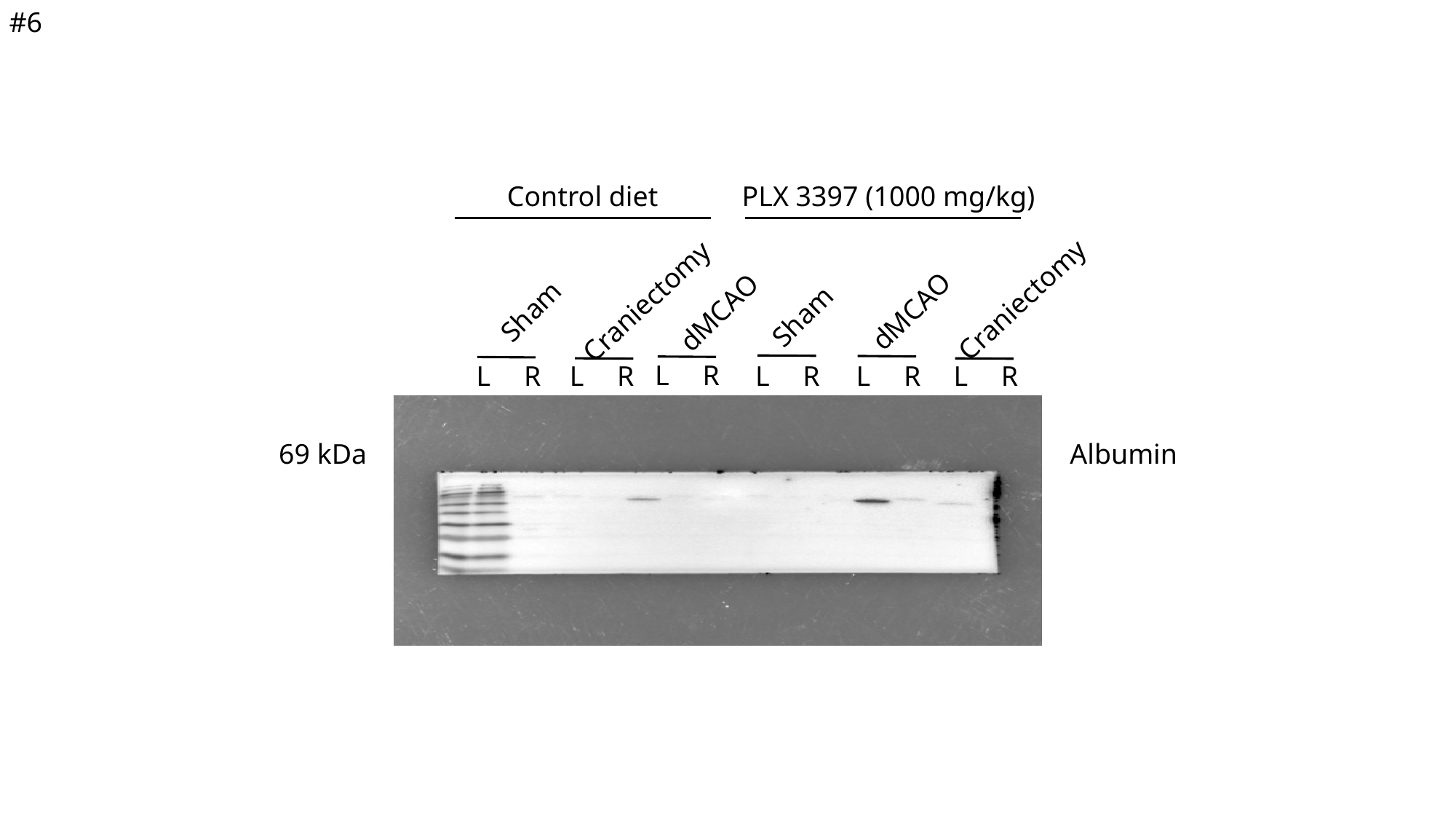

#6
Control diet
PLX 3397 (1000 mg/kg)
Craniectomy
Craniectomy
Sham
dMCAO
dMCAO
Sham
L
R
L
R
L
R
L
R
L
R
L
R
69 kDa
Albumin

## Slide 12
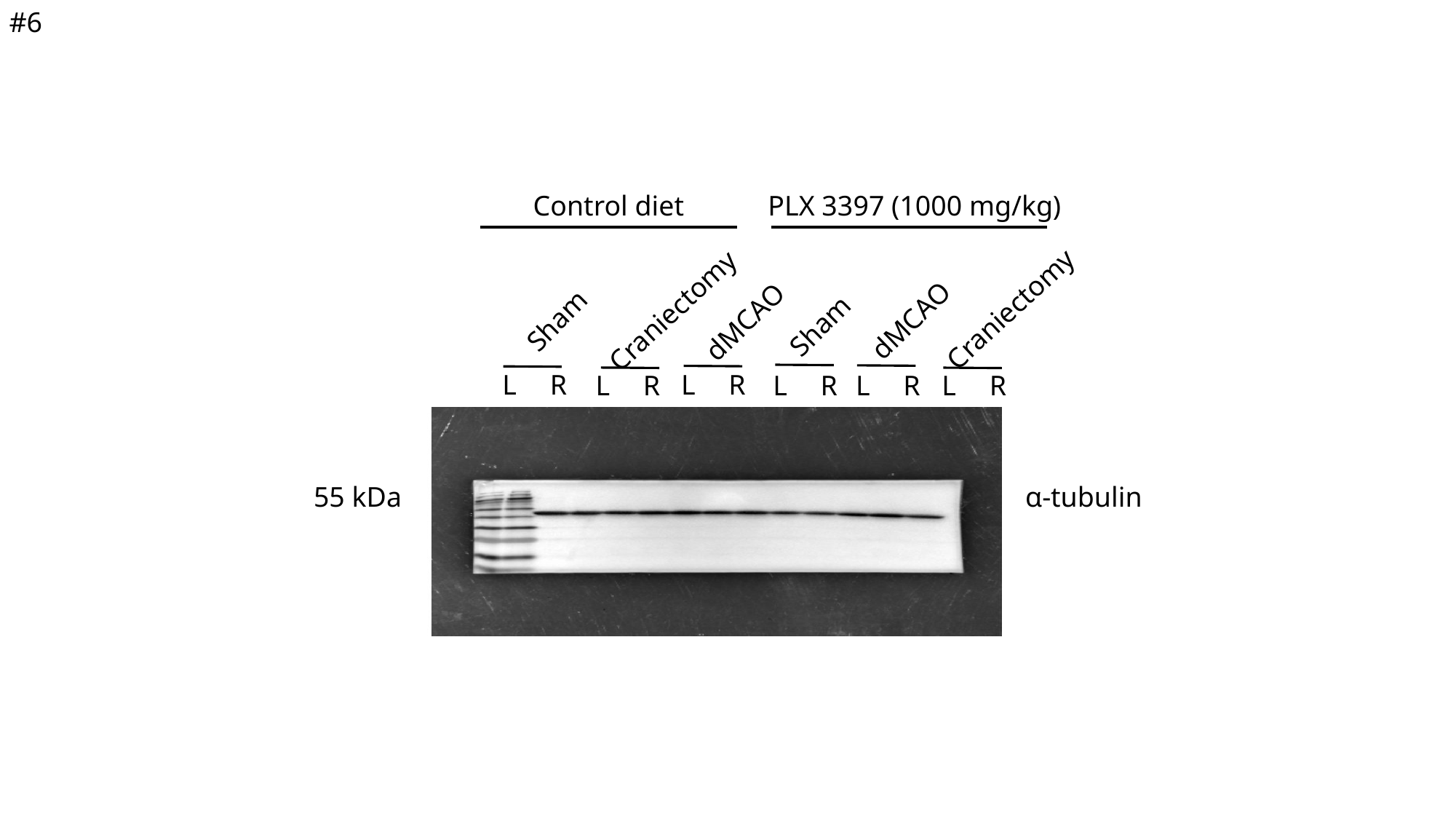

#6
Control diet
PLX 3397 (1000 mg/kg)
Craniectomy
Craniectomy
Sham
dMCAO
dMCAO
Sham
L
R
L
R
L
R
L
R
L
R
L
R
55 kDa
α-tubulin
